# CD8^+^ T Cell Immunity Is Compromised by Anti-CD20 Treatment and Rescued by Interleukin-17A

**DOI:** 10.1128/mBio.00447-20

**Published:** 2020-05-12

**Authors:** Facundo Fiocca Vernengo, Cristian G. Beccaria, Cintia L. Araujo Furlan, Jimena Tosello Boari, Laura Almada, Melisa Gorosito Serrán, Yamila Gazzoni, Carolina L. Montes, Eva V. Acosta Rodríguez, Adriana Gruppi

**Affiliations:** a Departamento de Bioquímica Clínica, Facultad de Ciencias Químicas (FCQ), Universidad Nacional de Córdoba (UNC), Córdoba, Argentina; b Centro de Investigaciones en Bioquímica Clínica e Inmunología (CIBICI—CONICET), Córdoba, Argentina; Max Planck Institute for Infection Biology; Stanford University

**Keywords:** anti-CD20, B lymphocytes, IL-17A, T cells, *Trypanosoma cruzi*

## Abstract

Treatment with anti-CD20, used in many diseases in which B cells play a pathogenic role, has been associated with susceptibility to intracellular infections. Here, we studied the effect of anti-CD20 injection on CD8^+^ T cell immunity using an experimental model of Trypanosoma cruzi infection, in which CD8^+^ T cells play a pivotal role. C57BL/6 mice were treated with anti-CD20 for B cell depletion prior to T. cruzi infection. Infected anti-CD20-treated mice exhibited a CD8^+^ T cell response with a conserved expansion phase followed by an early contraction, resulting in a strong reduction in total and parasite-specific CD8^+^ T cell numbers at 20 days postinfection. Anti-CD20 injection increased the frequency of apoptotic CD8^+^ T cells, decreased the number of effector and memory CD8^+^ T cells, and reduced the frequency of proliferating and cytokine-producing CD8^+^ T cells. Accordingly, infected anti-CD20-treated mice presented lower cytotoxicity of T. cruzi peptide-pulsed target cells *in vivo*. All of these alterations in CD8^+^ T cell immunity were associated with increased tissue parasitism. Anti-CD20 injection also dampened the CD8^+^ T cell response, when this had already been generated, indicating that B cells were involved in the maintenance rather than the induction of CD8^+^ T cell immunity. Anti-CD20 injection also resulted in a marked reduction in the frequency of interleukin-6 (IL-6)- and IL-17A-producing cells, and recombinant IL-17A (rIL-17A) injection partially restored the CD8^+^ T cell response in infected anti-CD20-treated mice. Thus, anti-CD20 reduced CD8^+^ T cell immunity, and IL-17A is a candidate for rescuing deficient responses either directly or indirectly.

## Abstract (precis)

Monoclonal antibody targeting the CD20 antigen on B cells is used to treat the majority of non-Hodgkin lymphoma patients and some autoimmune disorders. This therapy generates adverse effects, notably opportunistic infections and activation of viruses from latency. Here, using the infection murine model with the intracellular parasite Trypanosoma cruzi, we report that anti-CD20 treatment affects not only B cell responses but also CD8^+^ T cell responses, representing the most important immune effectors involved in control of intracellular pathogens. Anti-CD20 treatment, directly or indirectly, affects cytotoxic T cell number and function, and this deficient response was rescued by the cytokine IL-17A. The identification of IL-17A as the cytokine capable of reversing the poor response of CD8^+^ T cells provides information about a potential therapeutic treatment aimed at enhancing defective immunity induced by B cell depletion.

## INTRODUCTION

The anti-CD20 monoclonal antibody (mAb) has revolutionized the treatment of B cell malignancies. This antibody, which depletes B cells, has been a success for the treatment of non-Hodgkin’s lymphoma (1), chronic lymphocytic leukemia (2), and autoimmune disorders (3) and has also provided information about the antibody-independent role of B cells (4, 5).

B cells are known to produce antibodies (Abs), but they also take up, process, and present soluble antigens (Ags) and secrete cytokines. B cells were previously shown to produce interleukin-10 (IL-10) and to have regulatory functions in autoimmune models of colitis, experimental autoimmune encephalitis (EAE), and arthritis (6–8). However, B cells can produce cytokines other than IL-10. *Salmonella* triggers IL-35 and IL-10 production by B cells (9, 10), and we demonstrated previously that Trypanosoma cruzi infection leads B cells to produce IL-17A (11). In addition, B cells can produce transforming growth factor β1 (TGF-β1), and through this cytokine, they downregulated the function of antigen-presenting cells and encephalitogenic Th1/17 responses in a murine model of multiple sclerosis as previously shown (12). Therefore, under particular microenvironmental conditions, namely, through different activation and differentiation signals, B cells are able to produce different cytokines (13).

The role of B cells in conditioning CD8^+^ T cell responses has been reported in autoimmunity (14), in bacterial (15) and viral (16, 17) infections, and in cancer (18). B cells have been shown to shape the profile of CD8^+^ T cells, but the mediators involved in that process have not been completely elucidated.

In Chagas disease, which is caused by the protozoan parasite T. cruzi, CD8^+^ T cells capable of recognizing T. cruzi-infected cells are essential for control of the infection. Deletion or inhibition of CD8^+^ T cells results in uncontrollable parasite load early in infection and in an exacerbation of infection in chronically infected hosts (19, 20). Strategies that generate a productive specific CD8^+^ T cell response lead to increased host protection, a reduction in symptoms, and a decrease in disease transmission (21, 22). During T. cruzi infection, B cells undergo polyclonal expansion (23) and IL-17A production (11) and also regulate CD4^+^ T cell response (24). Considering these characteristics and the key functions of CD8^+^ T cells in controlling parasite replication, we used this experimental model to investigate how B cell depletion by anti-CD20 injection conditions CD8^+^ T cell immunity.

## RESULTS

### Anti-CD20 treatment decreased the number of CD8^+^ T cells and increased tissue parasitism.

**FIG 1:**
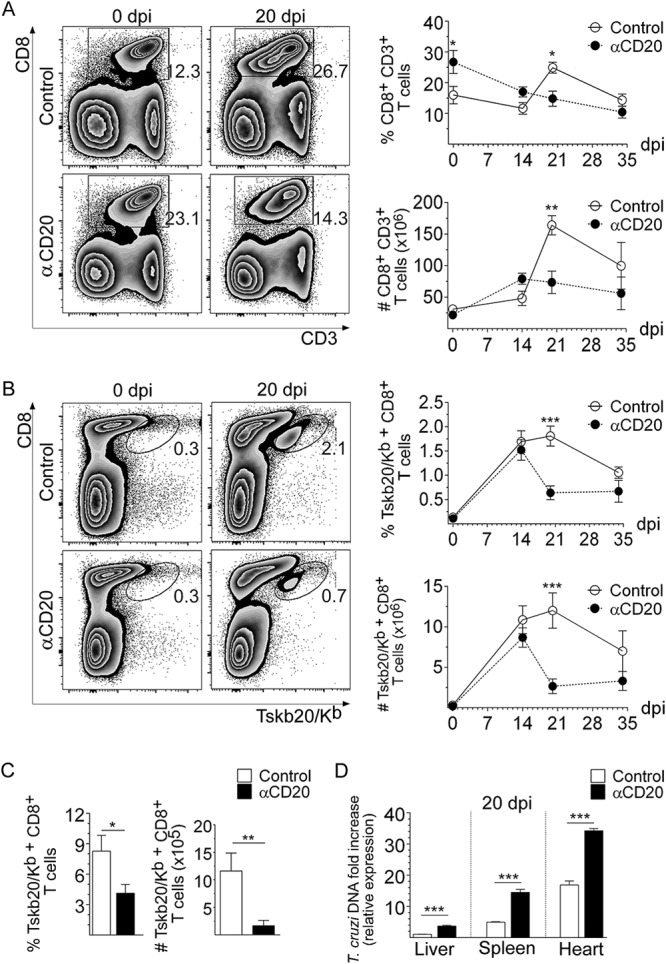
Anti-CD20 treatment reduced levels of CD8^+^ T cells and increased tissue parasitism. Mice injected with isotype control (control; in white circles) or anti-CD20 (αCD20; in black circles) mAb were infected with 5,000 trypomastigotes of T. cruzi strain Tulahuén. (A and B) Representative dot plot and statistical analysis of the means ± standard deviations (SD) of the percentage and number of (A) CD8^+^ CD3^+^ T cells and (B) Tskb20/K^b+^ CD8^+^ T cells, within the lymphocyte gate, in the spleen from uninfected (day 0) or infected mice at 20 dpi. Numbers within the plots indicate the frequency of cells in each region. (C) Statistical analysis of the mean ± SD of the percentage and number of Tskb20/K^b+^ CD8^+^ T cells in liver at 20 dpi in control (white bars) or anti-CD20-treated mice (black bars). (D) Relative amounts of T. cruzi satellite DNA in liver, spleen, and heart from infected control and anti-CD20-treated mice determined at 20 dpi. Murine GAPDH (glyceraldehyde-3-phosphate dehydrogenase) was used for normalization. Data are presented as means ± SD. Results are representative of four (A to C) and two (D) independent experiments with 4 to 5 mice per group each. *P* values were calculated with the two-tailed *t* test. *, *P* < 0.05; **, *P* < 0.01; ***, *P* < 0.001; n.s., not significant.

As reported by Tosello Boari et al. (25), we determined by flow cytometry that the frequency and number of CD8^+^ T cells increased during T. cruzi infection. CD8^+^ T cell expansion peaked at 20 days postinfection (dpi) and was followed by the contraction phase of the response (Fig. 1A, control mice). Anti-CD20 injection, 8 days prior to the infection, significantly decreased splenic B cell numbers (see Fig. S1A to C in the supplemental material), which persisted at very low levels until 20 dpi (Fig. S1B) and influenced CD8^+^ T cells (Fig. 1A to C). Anti-CD20 injection resulted in a significantly reduced CD8^+^ T cell frequency and number at 20 dpi (Fig. 1A, αCD20). As expected from the depletion of B cells, normal uninfected anti-CD20-treated mice had a higher frequency of CD8^+^ T cells than uninfected isotype-treated control mice (Fig. 1A, 0 dpi). The frequencies and numbers of splenic Tskb20-specific CD8^+^ T cells (parasite-specific CD8^+^ T cells) detected in infected control mice were similar to those detected in infected anti-CD20-treated mice until 14 dpi (Fig. 1B). After that, the percentage dropped dramatically in the anti-CD20-treated mice, which presented significantly lower frequencies and numbers of T. cruzi-specific CD8^+^ T cells at 20 dpi, remaining low up to 35 dpi (the last day of our analysis) (Fig. 1B). The frequency and number of Tskb20-specific CD8^+^ T cells were also reduced in liver, a T. cruzi infection target organ, of infected anti-CD20-treated mice at 20 dpi (Fig. 1C). In concordance with the low number of total and parasite-specific CD8^+^ T cells, anti-CD20-treated mice infected with T. cruzi had a higher parasite load than controls, as evidenced by the T. cruzi DNA fold increase measured in the liver, spleen, and heart at 20 dpi (Fig. 1D).

10.1128/mBio.00447-20.1 FIG S1 B cell depletion by anti-CD20 injection. (A) C57BL6 mice were injected with isotype control (control; in white bars) or anti-CD20 (in black bars) mAb, and B cell (CD19^+^B220^+^) frequency was determined in the spleen and blood at 8 days postinjection. (B and C) Mice injected with isotype control (control; in white circles) or anti-CD20 (in black circles) mAb were infected with 5,000 trypomastigotes of T. cruzi Tulahuén strain at 8 days after anti-CD20 injection. C57BL/6 untreated uninfected mice were processed in parallel (in gray). (B) Number of B cells determined by flow cytometry. Statistical differences were evaluated between infected control and anti-CD20-treated mice at different dpi. (C) Immunofluorescence of spleen sections (7 μm) from control and anti-CD20-treated mice at 14 dpi, stained with PE-labeled anti-B220 (white). Magnification: ×200. (Right) Statistical analysis of the percentage of area occupied by B220^+^ cells (*n* = 4 for infected control [white bar] or anti-CD20-treated [black bar] mice). *P* values were calculated with the two-tailed *t* test. Results are representative of two independent experiments with 4 to 6 mice per group each. Download FIG S1, TIF file, 2.4 MB. Copyright © 2020 Fiocca Vernengo et al. 2020 Fiocca Vernengo et al. This content is distributed under the terms of the Creative Commons Attribution 4.0 International license.

### Anti-CD20 treatment decreased the number of effector and memory CD8^+^ T cells and compromised their survival and proliferation.

**FIG 2:**
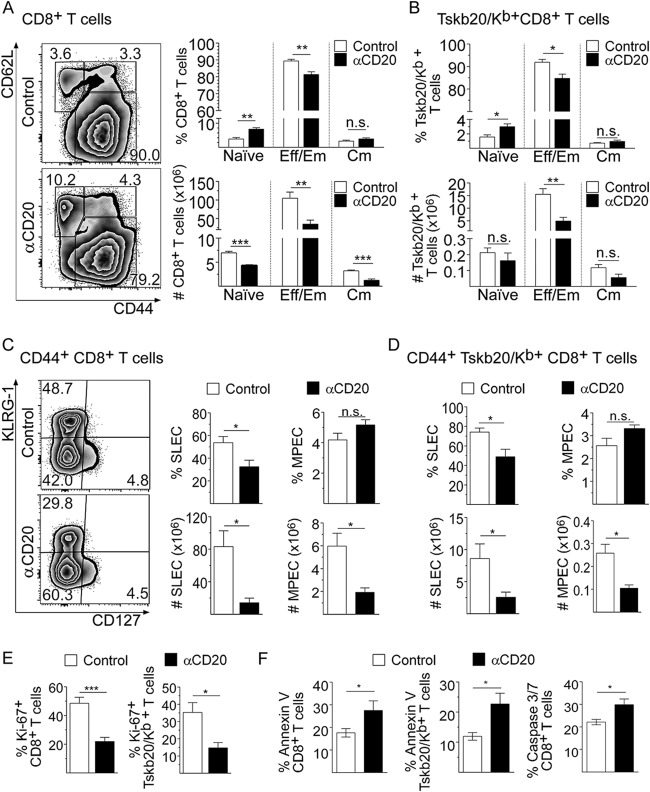
Anti-CD20 treatment decreased effector and memory CD8^+^ T cell number and compromised their survival and proliferation. Mice injected with isotype control (control; in white bars) or anti-CD20 (αCD20; in black bars) mAb were infected with 5,000 trypomastigotes of T. cruzi strain Tulahuén. Splenic cells were obtained at 20 dpi and analyzed by flow cytometry. (A) Representative plots of CD62L versus CD44 expression on CD8^+^ T cells. (A and B) Statistical analysis of the frequency and number of naive (CD62L^hi^ CD44^lo^), effector memory/effector (Eff/Em) (CD62L^lo^ CD44^hi^) and central memory (Cm) (CD62L^hi^ CD44^hi^) cells of (A) total CD8^+^ and (B) Tskb20/K^b+^ CD8^+^ T cells. (C) Representative plots of KLRG-1 versus CD127 expression on CD44^+^ CD8^+^ T cells. (C and D) Statistical analysis of the frequency and number of short-lived effector cells (SLEC: KLRG^hi^ CD127^lo^) and memory precursor effector cells (MPEC: KLRG1^lo^ CD127^hi^) of (C) total CD44^+^ CD8^+^ and (D) CD44^+^ Tskb20/K^b+^ CD8^+^ T cells. (E) Bar graphs representing the frequency of Ki-67^+^ cells on gated total CD8^+^ or Tskb20/K^b+^ CD8^+^ T cells. (F) Bar graphs representing the frequency of apoptotic annexin V^+^ 7ADD^neg^ cells in gated CD8^+^ or Tskb20/K^b+^ CD8^+^ T cells and active caspase 3/7^+^ Sytox^neg^ cells in total CD8^+^ T cells. Results are representative of three (A to D) and two (E and F) independent experiments with 4 to 5 mice per group each. All *P* values were calculated with the two-tailed *t* test.

An assessment of CD8^+^ T cell subset distribution, based on CD62L versus CD44 expression, showed that the spleen of infected anti-CD20-treated mice at 20 dpi presented a higher percentage of naive (CD62L^hi^ CD44^neg^) and lower percentages of effector memory/effector (CD62L^neg^ CD44^hi^) CD8^+^ T cells than their counterparts from infected control mice (Fig. 2A). Expressing the values in absolute numbers, we determined that anti-CD20-treated mice exhibited a significant reduction in the levels of all CD8^+^ T cell subpopulations (Fig. 2A). The anti-CD20 injection decreased the frequency and number of splenic Tskb20/K^b+^ CD8^+^ T cells with an effector phenotype but not the number of Tskb20/K^b+^ CD8^+^ T cells with a naive or central memory phenotype (Fig. 2B).

Considering that the general features of protective CD8^+^ T cell responses against intracellular pathogens consist of the generation and expansion of short-lived highly functional effector populations, we also evaluated the phenotype of CD8^+^ T cells based on KLRG-1 and CD127 expression (26). The frequency and number of total and parasite-specific CD8^+^ T cells with a short-lived effector cell (SLEC) phenotype (CD44^+^ KLRG1^hi^ CD127^lo^) were significantly reduced in the group of anti-CD20-treated mice at 20 dpi (Fig. 2C and D). The frequencies of total (Fig. 2C) and Tskb20/K^b+^ (Fig. 2D) CD8^+^ T cells with a memory precursor effector cell (MPEC) phenotype (CD44^+^ KLRG1^lo^ CD127^hi^) were similar in both experimental groups, but a strong reduction in the number of MPECs was observed in the infected anti-CD20-treated mice with respect to the infected control mice (Fig. 2C and D).

Splenic total and parasite-specific CD8^+^ T cells from infected anti-CD20-treated mice had a significantly lower frequency of proliferating Ki-67^+^ cells (Fig. 2E; see also Fig. S2A). In addition, a higher frequency of annexin V^+^/7-aminoactinomycin D-negative (7AAD^neg^) total and parasite-specific CD8^+^ T cells and active caspase 3/7^+^/Sytox^neg^ total CD8^+^ T cells was detected in infected anti-CD20-treated mice than in infected control mice (Fig. 2F; see also Fig. S2B), indicating that CD8^+^ T cells from B cell-depleted mice exhibited higher levels of apoptosis. Accordingly, a lower frequency of tetramethylrhodamine ethyl ester-high (TMRE^hi^) cells, as a quantification compatible with cellular viability, was detected in CD8^+^ T cells from infected anti-CD20-treated mice (Fig. S2C). Although there was a modest increase, we did not detect significant differences in the expression of the proapoptotic proteins BAD and Bim, determined as mean fluorescence intensity (MFI) in CD8^+^ T cells (Fig. S2D). Interestingly, we also observed a higher frequency of necrotic cells in the CD8^+^ T cell population in infected anti-CD20-treated mice in comparison to the infected controls (Fig. S2E). These results indicate that anti-CD20 injection (B cell depletion) partially arrested CD8^+^ T cell proliferation and favored CD8^+^ T cell death, leading to an early contraction of this protective response.

10.1128/mBio.00447-20.2 FIG S2 Frequency of proliferating and apoptotic cells. Mice infected with 5,000 trypomastigotes of T. cruzi strain Tulahuén were injected with isotype control mAb (control; white bars) or anti-CD20 mAb (black bars) 8 days before infection. (A) Representative dot plots of the frequency of Ki-67^+^ cells in total and of Tskb20/K^b+^ CD8^+^ T cells from infected control or anti-CD20-treated mice. (B) Representative dot plots of active caspase 3/7 and annexin V^+^ 7ADD^neg^ on gated CD8^+^ T cells from infected control or anti-CD20-treated mice. (C) Plots and bar graphs representing the frequency of viable nonapoptotic TMRE^hi^ cells on gated CD8^+^ or Tskb20/K^b+^ CD8^+^ T cells. Numbers within the plots indicate the frequency of cells in each region. (D) BAD and Bim expression determined by MFI in CD8^+^ T cells from infected control mAb-treated (white bars) or anti-CD20 mAb-treated (black bars) mice. (E) Frequency of necrotic cells (caspase 3/7^+^ Sytox^pos^) in gated CD8^+^ T cells from infected control mAb-treated (white bars) or anti-CD20 mAb-treated (black bars) mice. Bar graphs represent data as means ± SD. Results are representative of two independent experiments with 4 to 5 mice per group each. Download FIG S2, TIF file, 2.9 MB. Copyright © 2020 Fiocca Vernengo et al. 2020 Fiocca Vernengo et al. This content is distributed under the terms of the Creative Commons Attribution 4.0 International license.

### Anti-CD20 treatment resulted in lower CD8^+^ T cell functional activity.

**FIG 3:**
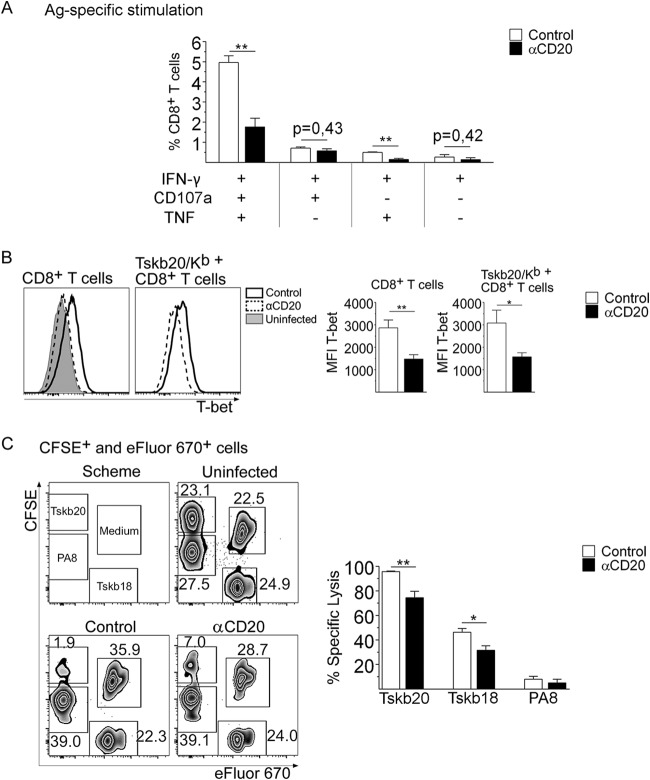
Anti-CD20 treatment resulted in lower CD8^+^ T cell functional activity. Mice injected with isotype control (control) or anti-CD20 (αCD20) mAb were infected with 5,000 trypomastigotes of T. cruzi strain Tulahuén. Uninfected mice were processed in parallel. Splenic cells were obtained at 20 dpi and analyzed by flow cytometry. (A) Bar graphs indicate the frequency ± SD of polyfunctional CD8^+^ T cells upon parasite antigen stimulation. Reference data corresponding to the different populations (IFN-γ^+^ TNF^+^ CD107a^+^ triple positive, IFN-γ^+^ TNF^+^ or IFN-γ^+^ CD107a^+^ double positive, and IFN-γ^+^ single positive CD8^+^ T cells) are indicated. (B) Representative histograms and statistical analysis of T-bet expression in total and Tskb20/K^b+^ CD8^+^ T cells from infected control (empty solid line) or anti-CD20-treated (empty dashed line) mice or uninfected mice (gray-filled solid line). (C) (Left) Representative dot plots of the frequency of transferred antigen-pulsed and unpulsed cells in the spleen of uninfected (top right) and infected (bottom left) control mice or anti-CD20-treated mice (bottom right) at 20 dpi. (Right) Statistical analysis of percentage of specific lysis in infected control (white bars) or anti-CD20-treated (black bars) mice. Results are representative of three (A and B) and two (C) independent experiments with 5 to 6 (A and B) or 4 to 5 (C) mice per group each. *P* values were calculated with the two-tailed *t* test.

In comparison to counterparts from infected control mice, splenic CD8^+^ T cells from infected anti-CD20-treated mice had a reduced frequency of polyfunctional CD8^+^ T cells (Fig. 3A), which are characterized by the simultaneous secretion of multiple cytokines and degranulation (see gating strategies in Fig. S3). Infected anti-CD20-treated mice exhibited a reduced frequency of gamma interferon-positive (IFN-γ^+^) tumor necrosis factor-positive (TNF^+^) CD107a^+^ (triple-positive) CD8^+^ T cells and IFN-γ^+^ TNF^+^ (double-positive) CD8^+^ T cells after *in vitro* stimulation with parasite peptide Tskb20 in comparison to infected control mice (Fig. 3A). Not only the polyfunctional CD8^+^ T cells were affected, but the frequency of total IFN-γ- or TNF-producing CD8^+^ T cells was also reduced in infected anti-CD20-treated mice (Fig. S4A; see Ag-specific stimulation data). In addition, splenocytes from infected anti-CD20-treated mice cultured with PMA (phorbol 12-myristate 13-acetate) and ionomycin had reduced frequencies of total IFN-γ^+^, TNF^+^, and CD107a^+^ and triple- and double-positive CD8^+^ T cells compared with splenocytes from controls (Fig. S4A and B; see polyclonal stimulation data). As highlighted by the reduced MFI values in the flow cytometry evaluation, we observed that Tskb20-specific CD8^+^ T cells had lower IFN-γ expression levels, indicating less IFN-γ production by parasite-specific CD8^+^ T cells from infected anti-CD20-treated mice (Fig. S4C). In agreement with the reduction in the frequency of IFN-γ-producing CD8^+^ T cells, total and parasite-specific CD8^+^ T cells from infected anti-CD20-treated mice exhibited lower T-bet expression than CD8^+^ T cells from control mice (Fig. 3B). Interestingly, CD8^+^ T cells from infected anti-CD20-treated mice had levels of T-bet similar to those measured in CD8^+^ T cells from uninfected mice (Fig. 3B; see histograms).

10.1128/mBio.00447-20.3 FIG S3 Flow cytometric gating strategy used to identify polyfunctional CD8^+^ T cells. Representative dot plots show the frequency of IFN-γ^+^, CD107a^+^, and TNF^+^ cells, gated on splenic CD8^+^ T cells, from infected control or anti-CD20-treated mice incubated with Medium or with PMA plus ionomycin (Polyclonal stimulation) or Tskb20 (Ag-specific stimulation) after 5 h of culture. Download FIG S3, TIF file, 2.9 MB. Copyright © 2020 Fiocca Vernengo et al. 2020 Fiocca Vernengo et al. This content is distributed under the terms of the Creative Commons Attribution 4.0 International license.

10.1128/mBio.00447-20.4 FIG S4 CD8^+^ T cell functionality after polyclonal and parasite-specific stimulation. (A) Statistical analysis of the frequency of total IFN-γ^+^, TNF^+^ or CD107a^+^ CD8^+^ T cells in the spleen of infected control (white bars) or anti-CD20-treated (black bars) mice obtained at 20 dpi and stimulated with PMA plus ionomycin (Polyclonal stimulation) or with Tskb20 (Ag-specific stimulation) after 5 h of culture. (B) Chart pie with the frequency ± SD of polyfunctional CD8^+^ T cells upon PMA plus ionomycin stimulation. References of the different populations (IFN-γ^+^ TNF^+^ CD107a^+^, triple positive; IFN-γ^+^ TNF^+^ or IFN-γ^+^ CD107a^+^, double positive; IFN-γ^+^ single positive CD8^+^ T cells) are indicated in the table at the right. (C) IFN-γ expression determined as MFI in CD8^+^ T cells in the spleen of infected control (white bars) or anti-CD20-treated (black bars) mice after *in vitro* Tskb20 stimulation. Data are presented as means ± SD. Results are representative of three independent experiments with 5 to 6 mice per group each. *P* values were calculated with the two-tailed *t* test. Download FIG S4, TIF file, 2.8 MB. Copyright © 2020 Fiocca Vernengo et al. 2020 Fiocca Vernengo et al. This content is distributed under the terms of the Creative Commons Attribution 4.0 International license.

Next, we evaluated the cytotoxic capacity elicited in infected anti-CD20-treated mice *in vivo* in comparison to that in infected control and uninfected mice. For the evaluation, differentially stained antigen-parasite-loaded cells were transferred to the different groups of mice. Panel C of Fig. 3 shows no specific lysis of PA8-loaded cells since this peptide is mainly present in other strains of the parasite, which are different with respect to the strain used in this study (27). In addition, the frequency of carboxyfluorescein succinimidyl ester-high (CFSE^hi^) eFluor670^neg^ Tskb20-pulsed cells and CFSE^neg^ eFluor670^+^ Tskb18-pulsed cells was higher in infected anti-CD20-treated mice than in infected controls. The results indicate that infected anti-CD20-treated mice displayed a reduced capacity to specifically kill target cells pulsed with the parasite antigens Tskb20 and Tskb18.

### Anti-CD20 treatment affected a developed CD8^+^ T cell response.

**FIG 4:**
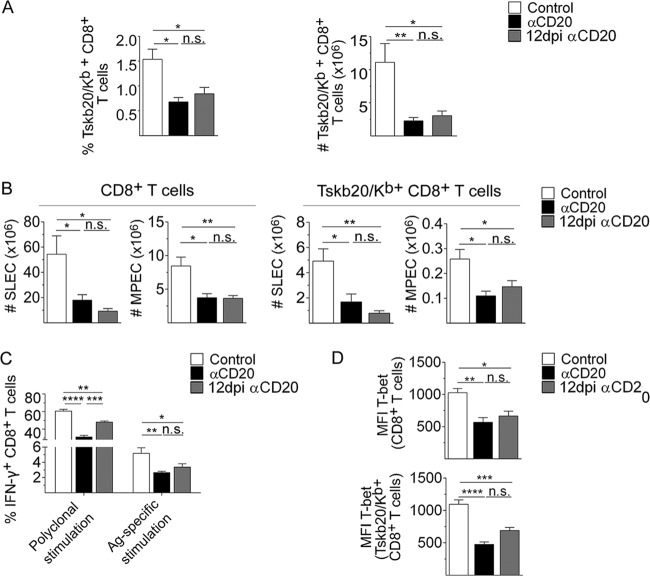
T. cruzi-infected mice treated with anti-CD20 mAb at 12 dpi also had a reduced CD8^+^ T cell response. Mice infected with 5,000 trypomastigotes of T. cruzi strain Tulahuén were injected with isotype control (control; white bars) or anti-CD20 mAb at 12 dpi (12 dpi αCD20; gray bars). Mice injected with anti-CD20 mAb 8 days before the infection with 5,000 trypomastigotes of T. cruzi strain Tulahuén were processed in parallel (αCD20; black bars). The spleens of the different groups of mice were obtained at 20 dpi. (A) Statistical analysis of the means ± SD of the percentages (left) and number (right) of Tskb20/K^b+^ CD8^+^ T cells. (B) Statistical analysis of the number of SLEC and MPEC in total CD8^+^ and Tskb20/K^b+^ CD8^+^ T cells. (C) Statistical analysis of total IFN-γ^+^ CD8^+^ T cell frequency of splenic cells stimulated with PMA plus ionomycin (Polyclonal stimulation) or with Tskb20 (Ag-specific stimulation) after 5 h of culture. ****, *P* < 0.0001. (D) Statistical analysis of T-bet expression on total and TSKB20/K^b+^ CD8^+^ T cells. Results are representative of three (A) and two (B to D) independent experiments with 5 to 6 (A) or 4 to 5 (B to D) mice per group each. *P* values were calculated with one-way ANOVA followed by Bonferroni’s posttest.

To analyze whether anti-CD20 injection affected an already generated CD8^+^ T cell response, T. cruzi-infected mice were injected with anti-CD20 mAb at 12 dpi, a time point at which the parasite-specific CD8^+^ T cell frequency had nearly peaked (see Fig. 1B). We determined that the injection of anti-CD20 at day 12 dpi induced a reduction in the frequency and number of splenic parasite-specific CD8^+^ T cells after 20 dpi, which was comparable with the effect of the treatment initiated before infection (Fig. 4A). Compared to infected control mice, mice treated with anti-CD20 after 12 dpi exhibited a significantly reduced number of CD8^+^ T cells with SLEC and MPEC phenotypes (Fig. 4B) and a diminished frequency of IFN-γ^+^ CD8^+^ T cells in splenocytes evaluated after polyclonal or antigen-specific stimulation (Fig. 4C). In line with the reduced frequency of IFN-γ-producing cells, total and parasite-specific CD8^+^ T cells from infected mice treated with anti-CD20 mAb at 12 dpi expressed lower levels of T-bet than CD8^+^ T cells from infected control mice (Fig. 4D). In our experimental model, the results indicated that anti-CD20 injection did not affect the induction of the CD8^+^ T cell response (see Fig. 1A) but probably affected their survival/maintenance.

### B cells from T. cruzi-infected mice produce cytokines involved in CD8^+^ T cell survival.

**FIG 5:**
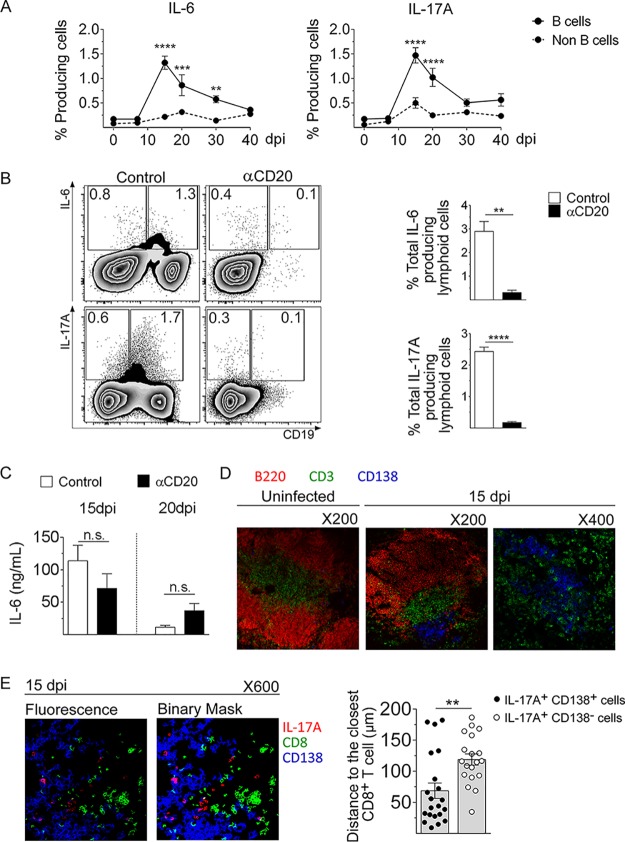
Splenic B cells from T. cruzi-infected mice were the main IL-6- and IL-17A-producing lymphoid cells. C57BL/6 mice were infected with 5,000 trypomastigotes of T. cruzi strain Tulahuén and analyzed at different dpi. Zero dpi indicates uninfected mice. (A) Statistical analysis of the percentage of IL-6-producing or IL-17A-producing CD19^+^ cells (B cells) or CD19^neg^ cells (Non B cells) within the lymphocyte gate, in spleen from uninfected (0 dpi) or infected mice at different dpi. (B) Representative plots (left) and statistical analysis (right) of IL-6- or IL-17A-producing lymphoid cells at 15 dpi, obtained from infected control (white bars) or anti-CD20-treated (black bars) mice. (C) Serum IL-6 concentration in infected control or αCD20-treated mice determined at 15 and 20 dpi. (D) Immunofluorescence of spleen sections from uninfected and T. cruzi-infected mice obtained at 15 dpi, stained with anti-CD3 (green), anti-B220 (red), and anti-CD138 (blue) antibodies. (E) Immunofluorescence of spleen sections from T. cruzi-infected mice obtained at 15 dpi, stained with anti-CD8 (green), anti-IL-17A (red), and anti-CD138 (blue) antibodies. The image on the right represents the binary expression of the positive fluorescence observed in the image on the left. The statistical analysis on the right represents the 360° nearest distance between an IL-17A-producing cell and the closest CD8^+^ T cell. Results are representative of three (A and B) and two (C to E) independent experiments with 4 to 5 (A to C) or 3 to 4 (D and E) mice per group each. *P* values were calculated with the two-tailed *t* test.

Cytokines contribute to the regulation of the contraction of the response, as well as to the long-term maintenance of memory CD8^+^ T cells (25, 28–30). On the basis of this and considering that anti-CD20 injection depletes B cells from mice, we hypothesize that B cells might be the source of cytokines involved in the maintenance of the CD8^+^ T cell response. By flow cytometry, we observed that T. cruzi-infected mice had IL-6- and IL-17A-producing B cells and that the numbers of these cells peaked at 15 dpi and remained high at 20 to 30 dpi (Fig. 5A). In comparison to lymphoid non-B cells, B cells were the main source of IL-6 and IL-17A (Fig. 5A), while the main sources of IL-10, IFN-γ, and TNF within the lymphoid population were non-B cells (Fig. S5A).

10.1128/mBio.00447-20.5 FIG S5 Source of IL-10, IFN-γ, and TNF in lymphoid splenic cells from T. cruzi-infected mice and spatial location of CD8^+^ T cells and IL-17A-producing cells. C57BL/6 mice were infected with 5,000 trypomastigotes of T. cruzi strain Tulahuén and evaluated at different dpi. Data corresponding to zero dpi indicate uninfected mice. (A) Statistical analysis of the percentages of IL-10-, IFN-γ-, and TNF-producing CD19^+^ (B) or CD19^neg^ (Non-B) cells within a lymphocyte gate in the spleen from uninfected or infected mice at different dpi. Data are presented as means ± SD. Results are representative of two independent experiments with 4 to 5 mice per group each. (B) Surface plot analysis representing spatial CD138 (blue), IL-17A (red), and CD8 (green) expression in the defined area (white dotted lines, 13.6 μm × 21.4 μm) from spleen of infected mice. Download FIG S5, TIF file, 2.9 MB. Copyright © 2020 Fiocca Vernengo et al. 2020 Fiocca Vernengo et al. This content is distributed under the terms of the Creative Commons Attribution 4.0 International license.

When mice were treated with anti-CD20 mAb prior to infection, significant decreases in the levels of IL-6- and IL-17A-producing lymphoid cells were observed (Fig. 5B). Most of the cytokine-producing B cells were CD19^low^, which is compatible with the plasmablast phenotype (11). Interestingly, anti-CD20 injection before the infection did not affect the serum IL-6 concentration since that IL-6 values were similar at 15 and 20 dpi (Fig. 5C). The IL-17A concentration was undetectable in the sera from both groups of infected mice (data not shown).

Next, by immunofluorescence, we evaluated the spatial distribution of splenic B cells, CD8^+^ T cells, and IL-17A-producing cells. Panel D of Fig. 5 shows B cell follicles (B220^+^) and CD3^+^ T cells in uninfected and infected control mice. As expected, the extrafollicular plasmablasts (CD138^+^) were located in the T cell zone only in the spleen of infected control mice (23). Interestingly, IL-17A-producing plasmablasts and other IL-17A-producing cells were found close to CD8^+^ T cells and the quantification of the distance between an IL-17A-producing cell and the closest CD8^+^ T cell showed that IL-17A-producing plasmablasts were significantly closer than other IL-17A-producing cells (Fig. 5E). To evaluate the spatial distribution of these populations, we performed a surface plot analysis of a selected area (Fig. S5B, white dotted line). As can be seen in the plot, expression of CD138 (blue) and that of IL-17A (red) colocalized in the same physical space and were, in turn, in intimate contact with the CD8 area (green) (Fig. S5B, right panel), suggesting a potential interaction/cross talk between IL-17A-producing cells and CD8^+^ T cells.

Interestingly, we observed that anti-CD20 injection previous to infection affected neither the frequency and number of total CD4^+^ T cells (Fig. S6A) nor T-bet expression in CD4^+^ T cells (Fig. S6B). However, anti-CD20 treatment in infected mice also decreased the frequency and number of IL-17A^+^ CD4^+^ T cells (Fig. S6C).

10.1128/mBio.00447-20.6 FIG S6 CD4^+^ T cell response in infected anti-CD20-treated mice. (A) Statistical analysis of the frequency and number of CD4^+^ T cells in the spleen of control (white bars) or anti-CD20-treated (black bars) mice analyzed 20 dpi with T. cruzi. (B) Statistical analysis of T-bet expression in CD4^+^ T cells in the spleen of control or anti-CD20-treated mice evaluated 20 dpi with T. cruzi. (C) Representative plots and statistical analysis of the frequency and number of splenic Th17 cells determined at 15 dpi in control and anti-CD20-treated mice, after stimulation with PMA plus ionomycin. Data are presented as means ± SD. Results are representative of two independent experiments with 5 to 6 mice per group each. *P* values were calculated with the two-tailed *t* test. Download FIG S6, TIF file, 2.3 MB. Copyright © 2020 Fiocca Vernengo et al. 2020 Fiocca Vernengo et al. This content is distributed under the terms of the Creative Commons Attribution 4.0 International license.

### Recombinant IL-17A, but not IL-6, partially restored the number and function of CD8^+^ T cells in infected anti-CD20-treated mice.

**FIG 6:**
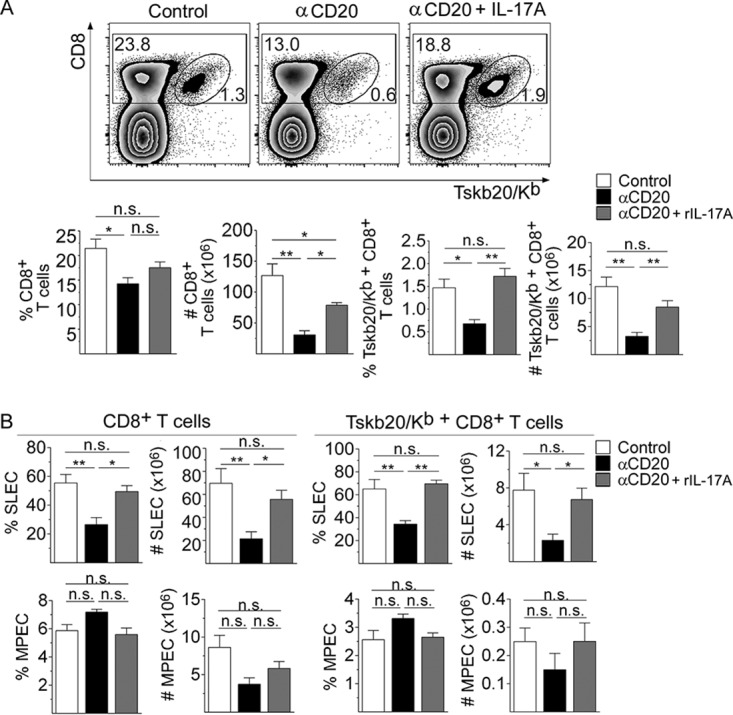
Recombinant IL-17A rescued the magnitude and effector phenotype of the CD8^+^ T cell response observed in infected anti-CD20-treated mice. Mice infected with 5,000 trypomastigotes of T. cruzi strain Tulahuén were injected with isotype control (control; white bars) or anti-CD20 mAb 8 days before infection. Infected mice injected with anti-CD20 mAb were also injected with PBS (αCD20, black bars) or rIL-17A (αCD20 + rIL-17A, gray bars) at 12, 14, 16, and 18 dpi. The spleens of the different groups of mice were obtained at 20 dpi. (A) (Top panels) Representative dot plot showing the percentage of total and TSKB20/K^b+^ CD8^+^ T cells, gated on lymphoid cells. (Bottom panels) Statistical analysis of the means ± SD of the percentages and number of indicated cells. (B) Statistical analysis of the number of SLEC and MPEC in total and Tskb20/K^b+^-specific CD8^+^ T cells. Results are representative of three independent experiments with 4 to 5 mice per group each. *P* values were calculated with one-way ANOVA followed by Bonferroni’s posttest.

To evaluate the hypothesis that the absence/diminution of IL-17A could affect the maintenance of the CD8^+^ T cell response in infected anti-CD20-treated mice, groups of these mice were injected with recombinant IL-17A (rIL-17A) or phosphate-buffered saline (PBS). Panel A of Fig. 6 shows that injections of rIL-17A partially restored the frequency and number of total and Tskb20-specific CD8^+^ T cells. In particular, the frequency and number of total and Tskb20-specific SLEC CD8^+^ T cells, but not the frequency and number of the MPEC CD8^+^ T cell population, were increased by rIL-17A supplementation (Fig. 6B).

**FIG 7:**
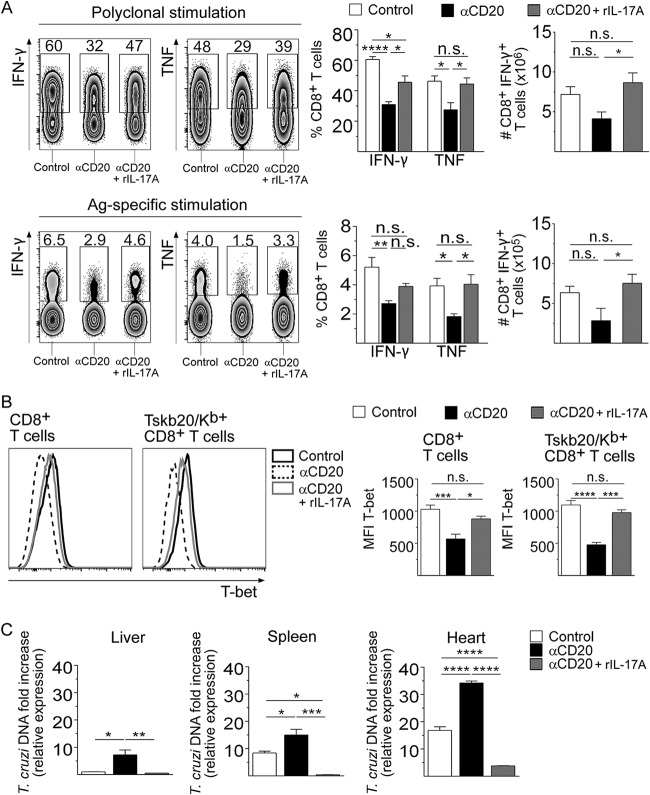
Recombinant IL-17A increased the functionality of CD8^+^ T cells and favored parasite control in infected anti-CD20-treated mice. Mice infected with 5,000 trypomastigotes of T. cruzi strain Tulahuén were injected with isotype control (control, white bars) or anti-CD20 mAb 8 days before infection. Infected mice injected with anti-CD20 mAb were injected with PBS (αCD20, black bars) or rIL-17A (αCD20 + rIL-17A, gray bars) at 12, 14, 16, and 18 dpi. The spleens of the different groups of mice were obtained at 20 dpi. (A) Representative dot plot and statistical analysis of the percentage and number of IFN-γ^+^ or the percentage of TNF^+^ cells, gated on CD8^+^ T cells, obtained after polyclonal or Ag-specific stimulation. (B) Representative histograms and statistical analysis of T-bet expression in total and Tskb20/K^b+^ CD8^+^ T cells from infected control (black solid line) or anti-CD20-treated mice injected with PBS (black dashed line) or with rIL-17A (gray solid line). (C) Relative amounts of T. cruzi satellite DNA in liver, spleen, and heart determined at 20 dpi. Murine GAPDH was used for normalization. Results are representative of three (A and B) and two (C) independent experiments with 5 to 6 (A and B) or 3 to 4 (C) mice per group each. *P* values were calculated with one-way ANOVA followed by Bonferroni’s posttest.

In addition, rIL-17A partially restored the frequency and number of IFN-γ-producing CD8^+^ T cells and the frequency of TNF-producing CD8^+^ T cells (Fig. 7A). The increase in the frequency of IFN-γ-producing CD8^+^ T cells in infected anti-CD20-treated mice injected with rIL-17A was accompanied by an increase in T-bet expression in total and Tskb20-specific CD8^+^ T cells (Fig. 7B). As expected, the increase in the number and functionality of CD8^+^ T cells in infected anti-CD20-treated mice injected with rIL-17A was associated with a strong reduction in the parasite load in the liver, spleen, and heart (Fig. 7C).

**FIG 8:**
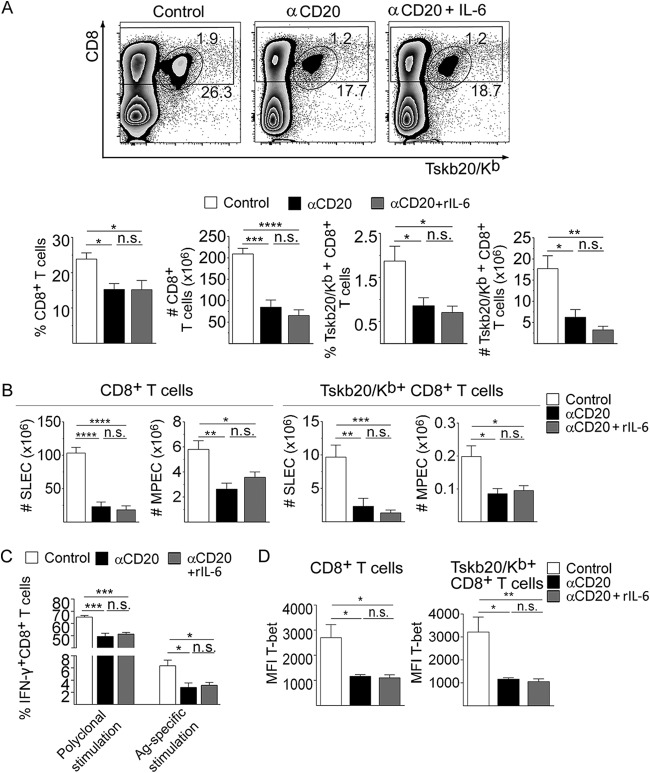
Recombinant IL-6 did not modify the magnitude and effector phenotype of the CD8^+^ T cell response observed in infected anti-CD20-treated mice. Mice infected with 5,000 trypomastigotes of T. cruzi strain Tulahuén were injected with isotype control (control; white bars) or anti-CD20 mAb 8 days before infection. Infected mice injected with anti-CD20 mAb were also injected with PBS (αCD20, black bars) or rIL-6 (αCD20 + rIL-6, gray bars) at 12, 14, 16, and 18 dpi. The spleens of the different groups of mice were obtained at 20 dpi. (A) (Top panels) Representative dot plot showing the percentage of total and TSKB20/K^b+^ CD8^+^ T cells, gated on lymphoid cells. (Bottom panels) Statistical analysis of the means ± SD of the percentages and number of indicated cells. (B) Statistical analysis of the number of SLEC and MPEC in total and Tskb20/K^b+^-specific CD8^+^ T cells. (C) Statistical analysis of total IFN-γ^+^ CD8^+^ T cell frequency of splenic cells stimulated with PMA plus ionomycin (Polyclonal stimulation) or with Tskb20 (Ag-specific stimulation) after 5 h of culture. (D) Statistical analysis of T-bet expression on total and TSKB20/K^b+^ CD8^+^ T cells. Results are representative of two independent experiments with 4 to 6 mice per group each. *P* values were calculated with one-way ANOVA followed by Bonferroni’s posttest.

Recombinant IL-6 injection in infected anti-CD20-treated mice did not increase the frequency and number of total and Tskb20-specific CD8^+^ T cells (Fig. 8A) and did not modify the frequency and number of SLECs and MPECs CD8^+^ T cell subsets (Fig. 8B). In addition, IL-6 injection into infected anti-CD20-treated mice was not able to modify total and Tskb20-specific IFN-γ production in CD8^+^ T cells (Fig. 8C) or T-bet expression in CD8^+^ T cells (Fig. 8D).

## DISCUSSION

An understanding of the effects of anti-CD20 treatment, which leads to the elimination of B cells, on other cell types allows the identification of different side effects of this therapy and highlights the Ab-independent functions of B cells. In this study, we found that anti-CD20 injection administered prior to the infection or after the time at which the specific CD8^+^ T cell response was developed altered antiparasitic CD8^+^ T cell immunity. In our work, we determined that the CD8^+^ T cell decrease in infected anti-CD20-treated mice affected the total number of CD8^+^ T cells and particularly that of SLEC. Interestingly, we did not observe an impact of anti-CD20 treatment on the frequency of memory CD8^+^ T cells. In fact, the data demonstrate no differences in the numbers of antigen-specific CD8^+^ T cells at 35 dpi. However, when the values representing the percentages of the different CD8^+^ T cell subsets were expressed in numbers, we observed a significant reduction in all of them as a consequence of the reduction in total CD8^+^ T cell numbers.

Our kinetics studies indicated that treatment with anti-CD20 prior to infection did not affect the induction phase of the CD8^+^ T cell response, indicating that B cells are not involved in the initial events that drive CD8^+^ T cell immunity during T. cruzi infection. Although the conclusions of our work were obtained using a single model, similar results were reported previously for Listeria monocytogenes infection (15), in which B cells did not play any role in the initial activation or microorganism-driven expansion of CD8^+^ T cells. Instead, we determined that, similarly to the L. monocytogenes infection model, depletion of B cells by anti-CD20 injection significantly accelerated the contraction phase of the CD8^+^ T cell response in T. cruzi-infected mice. Given the lower frequency of proliferating and viable (TMRE^hi^) CD8^+^ T cells and the higher frequency of apoptotic CD8^+^ T cells determined in infected anti-CD20-treated mice, it is likely that a reduced expansion rate, together with a lower survival rate, could be responsible for the early contraction of the CD8^+^ T cell response. In a similar way, a significant, long-lasting, and reversible depletion effect on CD8^+^ T cell counts was previously reported in patients with rheumatoid arthritis after 12 and 24 weeks of treatment with rituximab (31). Recently, it was reported that B cell depletion in patients with melanoma also reduced CD8^+^ T cell numbers and tumor-associated inflammation (32).

It was previously reported that B cells can tolerize CD8^+^ T cells (33), but we found that during T. cruzi infection, CD8^+^ T cells became less functional in the absence of B cells. Anti-CD20 treatment reduced the functionality of CD8^+^ T cells, as evidenced by a reduction in the frequency of cytokine-producing (IFN-γ or TNF) and polyfunctional (cytokine-producing and degranulating) CD8^+^ T cells. Accordingly, T-bet levels, determined as an indirect measure of IFN-γ production, were reduced in CD8^+^ T cells from anti-CD20-treated mice.

T. cruzi peptide-pulsed cell transfer demonstrated that infected anti-CD20-treated mice had a significantly reduced capability to lyse not only Tskb20-loaded target cells but also Tskb18-loaded cells *in vivo*, suggesting that this treatment affected not only the immunodominant CD8^+^ T cell response but also other T. cruzi-specific CD8^+^ T cell responses. Administration of the anti-CD20 treatment before or after infection led to an early contraction of the CD8^+^ T cell response, suggesting that B cells participate, either directly or indirectly, in the maintenance of CD8^+^ T cells.

Defective CD8^+^ T cell functional activity has been associated with increased pathogen load during chronic infections (34). The role of CD8^+^ T cells in the control of intracellular microorganisms was highlighted in patients infected with HIV, where the frequency and proportion of the HIV-specific T cell response with the highest functionality inversely correlated with the viral load in progressors (35). Accordingly, the deficient numbers and lower level of functionality of CD8^+^ T cells from infected anti-CD20-treated mice were found to be associated with a higher parasite load at 20 dpi.

Notably, it was previously reported that patients treated with anti-CD20 can develop different viral infections, which are the most common nonhematological adverse effects of this therapy. Viral infections in patients receiving anti-CD20 include severe respiratory tract infections, hepatitis B virus reactivation, and varicella-zoster virus infection (36–38). On the basis of our results, we hypothesize that treatment of patients with anti-CD20 not only affects Ab-secreting cells, which can neutralize viruses, but could also affect the development or establishment of CD8^+^ T cell responses, which are necessary for the control of intracellular infections. Additionally, we observed that anti-CD20 did not modify CD4^+^ T cell frequency and number but significantly affected IL-17A-producing CD4^+^ T cells. The results indicate that B cell depletion occurring, directly or indirectly, through the reduction in the levels of IL-17A-producing cells reduced CD8^+^ T cell immunity. Indeed, we observed that rIL-17A was able to partially reverse the deficient CD8^+^ T cell response.

In T. cruzi infection, two cytokines, IL-6 and IL-17A, have been reported to be involved in the improvement and maintenance of the CD8^+^ T cell response (25, 28). IL-6 improves the cytotoxic CD8^+^ T cell dysfunction triggered by nitric oxide in patients with Chagas disease (28); additionally, we recently demonstrated that IL-17RA and IL-17A are critical factors for sustaining CD8^+^ T cell immunity to T. cruzi (25). A possible role of IL-6 in the maintenance of the CD8^+^ T cell response in our experimental model was ruled out by the fact that anti-CD20 treatment did not modify the concentration of serum IL-6 in infected mice and that rIL-6 injection did not improve or restore the deficient CD8^+^ T cell response. In contrast, we observed that rIL-17A was able to reverse the quantitative and functional reduction in CD8^+^ T immunity observed in T. cruzi-infected anti-CD20-treated mice. The results reported here reinforce recent data obtained in our laboratory revealing the IL-17A/IL-17RA pathway-mediated roles of CD8^+^ T cells (25) and position IL-17A as a key cytokine in the maintenance of the cytotoxic T cell response. Our results are also supported by the findings reported by Acharya et al. (39), which showed that IL-17A directly potentiates CD8^+^ T cell cytotoxicity against West Nile virus infection.

In line with our findings, it was previously reported that μMT mice infected with T. cruzi strain Tulahuén exhibited a marked reduction in the CD8^+^ T cell subpopulation (40). Also, Sullivan and colleagues (41) observed that mice deficient in B cells that are infected with T. cruzi had a defective CD8^+^ T cell response. They postulated that specific antibodies are capable of restoring the deficient CD8^+^ T cell response, as passive transfer of serum from infected but not noninfected mice reversed the magnitude and functionality as well as the exhaustion phenotype observed in CD8^+^ T cells (41). Considering that transfer of purified antibodies was not performed in that study, it is possible that the observed effect might be associated with the presence of IL-17A in addition to the specific antibodies.

In this work, we have not established whether IL-17A acts, directly or indirectly, on CD8^+^ T cells. In line with a direct effect, we previously reported that CD8^+^ T cells upregulate the expression of the IL-17A receptor during T. cruzi infection and that IL-17A has a direct effect on CD8^+^ T cells (25). However, we cannot rule out the possibility that IL-17A also could have a direct effect on antigen-presenting cells (42). We observed that infected anti-CD20-treated and control mice had similar frequencies and numbers of dendritic cells and F4-80^+^ cells and that IL-17A treatment of infected anti-CD20-treated mice did not modify the quantity of these cell populations (data not shown). It was previously reported that IL-17A favors cytotoxic T cell responses against L. monocytogenes infection by enhancing dendritic cell cross presentation (44). Here, in our experimental model, we did not determine the functional activity of dendritic cells. Regardless of this limitation in our study, we found that the exogenous addition of IL-17A reversed the loss of number and functionality of CD8^+^ T cells in infected anti-CD20-treated mice.

Interestingly, we observed that IL-17A supplementation did not improve the CD8^+^ T cell response in infected control mice (data not shown). We hypothesize that baseline levels are sufficient to sustain the CD8^+^ T cell number and functionality and that only a sharp decrease (such as occurs in B cell-depleted mice) affects the response of the CD8^+^ T cell population.

In conclusion, our work provides evidence that anti-CD20 treatment affects not only B cell numbers but also IL-17A-producing cell numbers and CD8^+^ T cell responses. This knowledge may be relevant for the clinical management of patients with autoimmune diseases or lymphomas who are receiving anti-CD20 treatment. Furthermore, given that IL-17A was able to restore CD8^+^ T cell dysfunction in treated hosts, targeting the IL-17R pathway not only can help to control viral infections but also can help to fight against other microbes and, eventually, tumors.

## MATERIALS AND METHODS

### Mice, parasites, and experimental infection.

All animal experiments were approved by and conducted in accordance with the guidelines of the Institutional Animal Care and Use Committee of FCQ-UNC (Res. No. 854/18 CICUAL-FCQ). Female age-matched (2-to-3-month-old) mice were used. C57BL/6 wild-type (WT; JAX:000664) mice and μMT (B6.129S2-Ighmtm1Cgn/J; JAX:002288) mice were obtained from The Jackson Laboratories (USA) and housed in our animal facility. Mice were inoculated intraperitoneally (i.p.) with 5 × 10^3^ trypomastigotes of T. cruzi (Tulahuén strain)/0.2 ml PBS (45).

### Anti-CD20 injection.

Mice were injected i.p. with 50 μg of anti-CD20 mAb (Genentech; clone 5D2) or with mouse IgG2a control isotype (BioXcell; clone C1.18.4.) 8 days before infection with T. cruzi and studied at different days postinfection (dpi). In an alternative setting, mice were injected with anti-CD20 at 12 dpi and studied at 20 dpi.

### Quantification of parasite DNA.

Genomic DNA was purified from 50 μg of tissue using TRIzol reagent (Life Technologies) following the manufacturer’s instructions. Satellite DNA from T. cruzi (GenBank accession no. AY520036) was quantified by reverse transcription-PCR (RT-PCR) as previously reported (24).

### Cell preparation.

Blood, spleen, and liver-infiltrating cells were obtained as described previously (45).

### Antibodies and flow cytometry.

Cell suspensions were washed in PBS and incubated with LIVE/DEAD Fixable Cell Dead stain (Thermo Fisher Scientific) for 15 min at room temperature. Next, the cells were washed in ice-cold fluorescence-activated cell sorter (FACS) buffer (PBS–2% fetal bovine serum [FBS]) and incubated with fluorochrome-labeled Abs for 20 min at 4°C. Different combinations of the following anti-mouse Abs (Thermo Fisher Scientific, Biolegend) were used: fluorescein isothiocyanate (FITC)-labeled anti-CD8 (53-6.7) and anti-CD44 (IM7); phycoerythrin (PE)-labeled anti-CD127 (A7R34); peridinin chlorophyll protein (PerCp)-Cy5.5-labeled anti-CD19 (eBio1D3), anti-CD3 (145-2C11), and anti-CD62L (MEL-14); PECy7-labeled anti-B220 (RA3-6B2), anti-KLRG1 (2F1), and Alexa Fluor 647-labeled anti-CD8 (53-6.7); and allophycocyanin (APC)-eFluor 780-labeled anti-CD8 (53-6.7). T. cruzi-specific CD8^+^ T cells were evaluated using an APC-labeled tetramer of H-2K(b) molecules loaded with T. cruzi *trans*-sialidase immunodominant ANYKFTLV (Tskb20) peptide (NIH Tetramer Core Facility) (46). After staining, cells were washed and acquired in a FACSCanto II analyzer (BD Biosciences) and analyzed with FlowJo V10 software (TreesStar). Blood was directly incubated with the antibodies mentioned above, and erythrocytes were lysed with a 0.87% NH_4_Cl buffer prior to acquisition. Transcription factors (TF) were detected after cell fixation and permeabilization performed with a Foxp3 staining buffer set according to the protocol of the manufacturer (Thermo Fisher Scientific) using the following antibodies: PECy7-labeled anti-T-bet (4B10) and PE-labeled anti-Ki-67 (SolA15). For intracellular cytokine staining, cells were cultured for 5 h with 50 ng/ml PMA (phorbol 12-myristate 13-acetate) (Sigma), 1 μg/ml ionomycin (Sigma), brefeldin A (Thermo Fisher Scientific), and monensin (Thermo Fisher Scientific). Cells were fixed and permeabilized with BD Cytofix/Cytoperm and Perm/Wash (BD Biosciences) according to the manufacturer’s instructions. Cells were incubated with surface-staining antibodies and PE-labeled anti-IL-17A (eBio17B7), anti-IL-6 (MP5-20F3), APC-labeled anti-IFN-γ (XMG1.2), and anti-IL-10 (JES5-16E3).

### Immunofluorescence of spleen.

Spleens were collected and frozen over liquid nitrogen. Frozen sections 7 μm in thickness were cut, fixed for 10 min in cold acetone, left to dry at 25°C, and stored at −80°C until use. Slides were hydrated in Tris buffer and blocked for 30 min at 25°C with 10% normal mouse serum–Tris buffer. After blocking, slides were incubated for 50 min at 25°C with different combinations of the following anti-mouse Abs (Thermo Fisher Scientific, Biolegend, and BD Biosciences): Alexa Fluor 488-labeled anti-CD3 (HM3420) and anti-CD8 (53-6.7), PE-labeled anti-B220 (RA3-6B2) and anti-IL-17A (eBio17B7), and APC-labeled anti-CD138 (281-2). Slices were mounted with FluorSave (Merck Millipore). For immunofluorescent staining of intracellular IL-17A, tissue sections were prepared, fixed, permeabilized, and blocked using an Image-iT fixation/permeabilization kit (Invitrogen; catalog no. R37602) prior to IL-17A staining. Images were collected with an Olympus microscope (FV1000) and processed/analyzed using ImageJ64 1.52e (National Institutes of Health, USA). For B220^+^ area measurements and IL-17A expression analysis, we segmented cell objects by converting images into a binary mask using the default setting, after which standard built-in functions were used to segment out cell objects.

### Apoptosis.

Apoptosis of CD8^+^ T cells was assessed by double staining performed with a CellEvent caspase-3/7 green flow cytometry assay kit and SYTOX vital dye (Thermo Fisher Scientific, USA) and also by the use of annexin V FITC conjugate (BD Biosciences, USA) in combination with 7AAD staining following the manufacturer’s protocols.

Additionally, mitochondrial depolarization was measured by FACS analysis using 50 nM TMRE (Thermo Fisher Scientific) as described previously (47). The splenic cell suspensions were stained with anti-mouse CD8 and Tskb20/K^b^ tetramer prior to staining with CellEvent caspase-3/7 green detection reagents, annexin V, or TMRE. Nonapoptotic cells were defined as TMRE^hi^ within live single cells.

The levels of expression of proapoptotic BH3-only proteins Bid and BAD on total CD8^+^ T cells were determined also by flow cytometry using anti-mouse Bim (clone K.912.7) and BAD (clone SA32-01) followed by goat anti-rabbit IgG (all from Invitrogen).

### CD8^+^ T cell effector function *in vitro*.

CD8^+^ T cell effector function was determined *in vitro* by CD107a mobilization and cytokine production, as previously reported (25). Briefly, cell suspensions were cultured for 5 h with medium, 5 μg/ml TSKB20 (ANYKFTLV) peptide (Genscript Inc.), or 50 ng/ml PMA plus 500 ng/ml ionomycin (Sigma) in the presence of monensin (Thermo Fisher Scientific) and a PE-labeled anti-CD107a mAb (Thermo Fisher Scientific; eBio1D4B). After culture, the cells were stained with a PECy7-labeled anti-CD8 mAb, fixed, and permeabilized with BD Cytofix/Cytoperm and Perm/Wash (BD Biosciences) according to the manufacturer’s instructions. After permeabilization, the cells were incubated for 30 min at room temperature with the following anti-mouse Abs (Thermo Fisher Scientific): APC-labeled anti-IFN-γ (XMG1.2) and PerCp-Cy5.5-labeled anti-TNF (MP6-XT22).

### *In vivo* cytotoxicity assay.

Spleen mononuclear cell suspensions from μMT mice were pulsed for 1 h with 1 μg/ml of the Tskb20, Tskb18, or PA8 (VNHRFTLV) peptides (target cells). In this setting, μMT mice, which lack mature B cells, were used to avoid circulating anti-CD20 killing of the transferred cells. Unpulsed splenocytes were used as a control. Target and control cells were washed and stained with a 2 μM or 20 μM concentration of CFSE and a 5 μM concentration of eFluor 670 cell proliferation dye (Thermo Fisher Scientific). After staining, cells were mixed in equal proportions and injected intravenously (i.v.) into uninfected and infected control and anti-CD20-treated mice at 20 dpi. Mice were sacrificed 5 h later, and the frequency of injected cells in the spleen was evaluated by flow cytometry. The differences in the percentages of specific lysis between the unloaded cells and each of the different peptide-loaded cell populations were calculated independently as follows: 100 — {[(percent peptide pulsed in infected cells/percent peptide unpulsed in infected cells)/(percent peptide pulsed in uninfected cells/percent peptide unpulsed in uninfected cells)] × 100}.

### Cytokine quantification.

Serum IL-6 and IL-17A concentrations were assessed by enzyme-linked immunosorbent assay (ELISA) according to the instructions of the manufacturer (Thermo Fisher Scientific).

### *In vivo* IL-6 and IL-17A treatment.

Anti-CD20-treated mice were administered with recombinant IL-6 or IL-17A, taking into account their production kinetics (Fig. 5A). Either IL-6 (200 ng/dose) (48) or IL-17A (500 ng/dose) (49) (Shenandoah Biotechnology, USA) was injected intraperitoneally at 12, 14, 16, and 18 dpi. Anti-CD20-treated mice injected with PBS were used as the non-cytokine-treated control group.

### Statistics.

The statistical significance of comparisons of mean values was assessed as indicated by a two-tailed Student's *t* test and two-way analysis of variance (ANOVA) followed by Bonferroni’s posttest using GraphPad software.

## References

[1] Coiffier B, Thieblemont C, Van Den Neste E, Lepeu G, Plantier I, Castaigne S, Lefort S, Marit G, Macro M, Sebban C, Belhadj K, Bordessoule D, Fermé C, Tilly H 2010 Long-term outcome of patients in the LNH-98.5 trial, the first randomized study comparing rituximab-CHOP to standard CHOP chemotherapy in DLBCL patients: a study by the Groupe d’Etudes des Lymphomes de l’Adulte. Blood 116:2040–2045. doi:10.1182/blood-2010-03-276246. 20548096 PMC2951853

[2] Hallek M, Fischer K, Fingerle-Rowson G, Fink AM, Busch R, Mayer J, Hensel M, Hopfinger G, Hess G, von Grünhagen U, Bergmann M, Catalano J, Zinzani PL, Caligaris-Cappio F, Seymour JF, Berrebi A, Jäger U, Cazin B, Trneny M, Westermann A, Wendtner CM, Eichhorst BF, Staib P, Bühler A, Winkler D, Zenz T, Böttcher S, Ritgen M, Mendila M, Kneba M, Döhner H, Stilgenbauer S, International Group of Investigators; German Chronic Lymphocytic Leukaemia Study Group. 2010 Addition of rituximab to fludarabine and cyclophosphamide in patients with chronic lymphocytic leukaemia: a randomised, open-label, phase 3 trial. Lancet 376:1164–1174. doi:10.1016/S0140-6736(10)61381-5. 20888994

[3] Edwards JCW, Szczepański L, Szechiński J, Filipowicz-Sosnowska A, Emery P, Close DR, Stevens RM, Shaw T 2004 Efficacy of B-cell-targeted therapy with rituximab in patients with rheumatoid arthritis. N Engl J Med 350:2572–2581. doi:10.1056/NEJMoa032534. 15201414

[4] Kelly-Scumpia KM, Scumpia PO, Weinstein JS, Delano MJ, Cuenca AG, Nacionales DC, Wynn JL, Lee PY, Kumagai Y, Efron PA, Akira S, Wasserfall C, Atkinson MA, Moldawer LL 2011 B cells enhance early innate immune responses during bacterial sepsis. J Exp Med 208:1673–1682. doi:10.1084/jem.20101715. 21746813 PMC3149216

[5] Elsegeiny W, Eddens T, Chen K, Kolls JK 2015 Anti-CD20 antibody therapy and susceptibility to Pneumocystis pneumonia. Infect Immun 83:2043–2052. doi:10.1128/IAI.03099-14. 25733518 PMC4399075

[6] Mizoguchi A, Mizoguchi E, Takedatsu H, Blumberg RS, Bhan AK 2002 Chronic intestinal inflammatory condition generates IL-10-producing regulatory B cell subset characterized by CD1d upregulation. Immunity 16:219–230. doi:10.1016/s1074-7613(02)00274-1. 11869683

[7] Fillatreau S, Sweenie CH, McGeachy MJ, Gray D, Anderton SM 2002 B cells regulate autoimmunity by provision of IL-10. Nat Immunol 3:944–950. doi:10.1038/ni833. 12244307

[8] Mauri C, Gray D, Mushtaq N, Londei M 2003 Prevention of arthritis by interleukin 10-producing B cells. J Exp Med 197:489–501. doi:10.1084/jem.20021293. 12591906 PMC2193864

[9] Neves P, Lampropoulou V, Calderon-Gomez E, Roch T, Stervbo U, Shen P, Kuhl AA, Loddenkemper C, Haury M, Nedospasov SA, Kaufmann SH, Steinhoff U, Calado DP, Fillatreau S 2010 Signaling via the MyD88 adaptor protein in B cells suppresses protective immunity during Salmonella typhimurium infection. Immunity 33:777–790. doi:10.1016/j.immuni.2010.10.016. 21093317

[10] Shen P, Roch T, Lampropoulou V, O'Connor RA, Stervbo U, Hilgenberg E, Ries S, Dang VD, Jaimes Y, Daridon C, Li R, Jouneau L, Boudinot P, Wilantri S, Sakwa I, Miyazaki Y, Leech MD, McPherson RC, Wirtz S, Neurath M, Hoehlig K, Meinl E, Grützkau A, Grün JR, Horn K, Kühl AA, Dörner T, Bar-Or A, Kaufmann SHE, Anderton SM, Fillatreau S 2014 IL-35-producing B cells are critical regulators of immunity during autoimmune and infectious diseases. Nature 507:366–370. doi:10.1038/nature12979. 24572363 PMC4260166

[11] Bermejo DA, Jackson SW, Gorosito-Serran M, Acosta-Rodriguez EV, Amezcua-Vesely MC, Sather BD, Singh AK, Khim S, Mucci J, Liggitt D, Campetella O, Oukka M, Gruppi A, Rawlings DJ 2013 Trypanosoma cruzi trans-sialidase initiates a program independent of the transcription factors RORgammat and Ahr that leads to IL-17 production by activated B cells. Nat Immunol 14:514–522. doi:10.1038/ni.2569. 23563688 PMC3631452

[12] Bjarnadottir K, Benkhoucha M, Merkler D, Weber MS, Payne NL, Bernard CC, Molnarfi N, Lalive PH 2016 B cell-derived transforming growth factor-beta1 expression limits the induction phase of autoimmune neuroinflammation. Sci Rep 6:34594. doi:10.1038/srep34594. 27708418 PMC5052622

[13] Vazquez MI, Catalan-Dibene J, Zlotnik A 2015 B cells responses and cytokine production are regulated by their immune microenvironment. Cytokine 74:318–326. doi:10.1016/j.cyto.2015.02.007. 25742773 PMC4475485

[14] Guo L, Kapur R, Aslam R, Speck ER, Zufferey A, Zhao Y, Kim M, Lazarus AH, Ni H, Semple JW 2016 CD20+ B-cell depletion therapy suppresses murine CD8+ T-cell-mediated immune thrombocytopenia. Blood 127:735–738. doi:10.1182/blood-2015-06-655126. 26556550

[15] Shen H, Whitmire JK, Fan X, Shedlock DJ, Kaech SM, Ahmed R 2003 A specific role for B cells in the generation of CD8 T cell memory by recombinant Listeria monocytogenes. J Immunol 170:1443–1451. doi:10.4049/jimmunol.170.3.1443. 12538706

[16] Lykken JM, DiLillo DJ, Weimer ET, Roser-Page S, Heise MT, Grayson JM, Weitzmann MN, Tedder TF 2014 Acute and chronic B cell depletion disrupts CD4+ and CD8+ T cell homeostasis and expansion during acute viral infection in mice. J Immunol 193:746–756. doi:10.4049/jimmunol.1302848. 24928986 PMC4290158

[17] Asano MS, Ahmed R 1996 CD8 T cell memory in B cell-deficient mice. J Exp Med 183:2165–2174. doi:10.1084/jem.183.5.2165. 8642326 PMC2192575

[18] DiLillo DJ, Yanaba K, Tedder TF 2010 B cells are required for optimal CD4+ and CD8+ T cell tumor immunity: therapeutic B cell depletion enhances B16 melanoma growth in mice. J Immunol 184:4006–4016. doi:10.4049/jimmunol.0903009. 20194720 PMC3733120

[19] Tarleton RL 2007 Immune system recognition of Trypanosoma cruzi. Curr Opin Immunol 19:430–434. doi:10.1016/j.coi.2007.06.003. 17651955

[20] Fernandes MC, Andrews NW 2012 Host cell invasion by Trypanosoma cruzi: a unique strategy that promotes persistence. FEMS Microbiol Rev 36:734–747. doi:10.1111/j.1574-6976.2012.00333.x. 22339763 PMC3319478

[21] Jordan KA, Hunter CA 2010 Regulation of CD8+ T cell responses to infection with parasitic protozoa. Exp Parasitol 126:318–325. doi:10.1016/j.exppara.2010.05.008. 20493842 PMC2934887

[22] Padilla AM, Simpson LJ, Tarleton RL 2009 Insufficient TLR activation contributes to the slow development of CD8+ T cell responses in Trypanosoma cruzi infection. J Immunol 183:1245–1252. doi:10.4049/jimmunol.0901178. 19553540

[23] Bermejo DA, Amezcua Vesely MC, Khan M, Acosta Rodriguez EV, Montes CL, Merino MC, Toellner KM, Mohr E, Taylor D, Cunningham AF, Gruppi A 2011 Trypanosoma cruzi infection induces a massive extrafollicular and follicular splenic B-cell response which is a high source of non-parasite-specific antibodies. Immunology 132:123–133. doi:10.1111/j.1365-2567.2010.03347.x. 20875075 PMC3015082

[24] Gorosito Serrán M, Tosello Boari J, Fiocca Vernengo F, Beccaria CG, Ramello MC, Bermejo DA, Cook AG, Vinuesa CG, Montes CL, Acosta Rodriguez EV, Gruppi A 2017 Unconventional pro-inflammatory CD4(+) T cell response in B cell-deficient mice infected with Trypanosoma cruzi. Front Immunol 8:1548. doi:10.3389/fimmu.2017.01548. 29209313 PMC5702327

[25] Tosello Boari J, Araujo Furlan CL, Fiocca Vernengo F, Rodriguez C, Ramello MC, Amezcua Vesely MC, Gorosito Serrán M, Nunez NG, Richer W, Piaggio E, Montes CL, Gruppi A, Acosta Rodriguez EV 2018 IL-17RA-signaling modulates CD8+ T cell survival and exhaustion during Trypanosoma cruzi infection. Front Immunol 9:2347. doi:10.3389/fimmu.2018.02347. 30364284 PMC6193063

[26] Joshi NS, Cui W, Chandele A, Lee HK, Urso DR, Hagman J, Gapin L, Kaech SM 2007 Inflammation directs memory precursor and short-lived effector CD8(+) T cell fates via the graded expression of T-bet transcription factor. Immunity 27:281–295. doi:10.1016/j.immuni.2007.07.010. 17723218 PMC2034442

[27] Low HP, Santos MA, Wizel B, Tarleton RL 1998 Amastigote surface proteins of Trypanosoma cruzi are targets for CD8+ CTL. J Immunol 160:1817–1823. 9469442

[28] Sanmarco LM, Visconti LM, Eberhardt N, Ramello MC, Ponce NE, Spitale NB, Vozza ML, Bernardi GA, Gea S, Minguez AR, Aoki MP 2016 IL-6 improves the nitric oxide-induced cytotoxic CD8+ T cell dysfunction in human Chagas disease. Front Immunol 7:626. doi:10.3389/fimmu.2016.00626. 28066435 PMC5179535

[29] Badovinac VP, Porter BB, Harty JT 2004 CD8+ T cell contraction is controlled by early inflammation. Nat Immunol 5:809–817. doi:10.1038/ni1098. 15247915

[30] Cousens LP, Peterson R, Hsu S, Dorner A, Altman JD, Ahmed R, Biron CA 1999 Two roads diverged: interferon alpha/beta- and interleukin 12-mediated pathways in promoting T cell interferon gamma responses during viral infection. J Exp Med 189:1315–1328. doi:10.1084/jem.189.8.1315. 10209048 PMC2193028

[31] Mélet J, Mulleman D, Goupille P, Ribourtout B, Watier H, Thibault G 2013 Rituximab-induced T cell depletion in patients with rheumatoid arthritis: association with clinical response. Arthritis Rheum 65:2783–2790. doi:10.1002/art.38107. 23918413

[32] Griss J, Bauer W, Wagner C, Simon M, Chen M, Grabmeier-Pfistershammer K, Maurer-Granofszky M, Roka F, Penz T, Bock C, Zhang G, Herlyn M, Glatz K, Laubli H, Mertz KD, Petzelbauer P, Wiesner T, Hartl M, Pickl WF, Somasundaram R, Steinberger P, Wagner SN 2019 B cells sustain inflammation and predict response to immune checkpoint blockade in human melanoma. Nat Commun 10:4186. doi:10.1038/s41467-019-12160-2. 31519915 PMC6744450

[33] Bennett SR, Carbone FR, Toy T, Miller JF, Heath WR 1998 B cells directly tolerize CD8(+) T cells. J Exp Med 188:1977–1983. doi:10.1084/jem.188.11.1977. 9841912 PMC2212383

[34] Mueller SN, Ahmed R 2009 High antigen levels are the cause of T cell exhaustion during chronic viral infection. Proc Natl Acad Sci U S A 106:8623–8628. doi:10.1073/pnas.0809818106. 19433785 PMC2688997

[35] Betts MR, Nason MC, West SM, De Rosa SC, Migueles SA, Abraham J, Lederman MM, Benito JM, Goepfert PA, Connors M, Roederer M, Koup RA 2006 HIV nonprogressors preferentially maintain highly functional HIV-specific CD8+ T cells. Blood 107:4781–4789. doi:10.1182/blood-2005-12-4818. 16467198 PMC1895811

[36] Aksoy S, Harputluoglu H, Kilickap S, Dede DS, Dizdar O, Altundag K, Barista I 2007 Rituximab-related viral infections in lymphoma patients. Leuk Lymphoma 48:1307–1312. doi:10.1080/10428190701411441. 17613758

[37] Cohen SB, Emery P, Greenwald MW, Dougados M, Furie RA, Genovese MC, Keystone EC, Loveless JE, Burmester GR, Cravets MW, Hessey EW, Shaw T, Totoritis MC; REFLEX Trial Group. 2006 Rituximab for rheumatoid arthritis refractory to anti-tumor necrosis factor therapy: results of a multicenter, randomized, double-blind, placebo-controlled, phase III trial evaluating primary efficacy and safety at twenty-four weeks. Arthritis Rheum 54:2793–2806. doi:10.1002/art.22025. 16947627

[38] Hua Q, Zhu Y, Liu H 2015 Severe and fatal adverse events risk associated with rituximab addition to B-cell non-Hodgkin’s lymphoma (B-NHL) chemotherapy: a meta-analysis. J Chemother 27:365–370. doi:10.1179/1973947815Y.0000000025. 25872413

[39] Acharya D, Wang P, Paul AM, Dai J, Gate D, Lowery JE, Stokic DS, Leis AA, Flavell RA, Town T, Fikrig E, Bai F 16 12 2017, posting date Interleukin-17A promotes CD8+ T cell cytotoxicity to facilitate West Nile virus clearance. J Virol doi:10.1128/JVI.01529-16. PMC5165211 27795421

[40] Cardillo F, Postol E, Nihei J, Aroeira LS, Nomizo A, Mengel J 2007 B cells modulate T cells so as to favour T helper type 1 and CD8+ T-cell responses in the acute phase of Trypanosoma cruzi infection. Immunology 122:584–595. doi:10.1111/j.1365-2567.2007.02677.x. 17635611 PMC2266037

[41] Sullivan NL, Eickhoff CS, Sagartz J, Hoft DF 2015 Deficiency of antigen-specific B cells results in decreased Trypanosoma cruzi systemic but not mucosal immunity due to CD8 T cell exhaustion. J Immunol 194:1806–1818. doi:10.4049/jimmunol.1303163. 25595788 PMC4324165

[42] Nakai K, He YY, Nishiyama F, Naruse F, Haba R, Kushida Y, Katsuki N, Moriue T, Yoneda K, Kubota Y 2017 IL-17A induces heterogeneous macrophages, and it does not alter the effects of lipopolysaccharides on macrophage activation in the skin of mice. Sci Rep 7:12473. doi:10.1038/s41598-017-12756-y. 28963556 PMC5622065

[43] Reference deleted.

[44] Xu S, Han Y, Xu X, Bao Y, Zhang M, Cao X 2010 IL-17A-producing gammadeltaT cells promote CTL responses against Listeria monocytogenes infection by enhancing dendritic cell cross-presentation. J Immunol 185:5879–5887. doi:10.4049/jimmunol.1001763. 20956351

[45] Tosello Boari J, Amezcua Vesely MC, Bermejo DA, Ramello MC, Montes CL, Cejas H, Gruppi A, Acosta Rodríguez EV 2012 IL-17RA signaling reduces inflammation and mortality during Trypanosoma cruzi infection by recruiting suppressive IL-10-producing neutrophils. PLoS Pathog 8:e1002658. doi:10.1371/journal.ppat.1002658. 22577359 PMC3343119

[46] Martin DL, Weatherly DB, Laucella SA, Cabinian MA, Crim MT, Sullivan S, Heiges M, Craven SH, Rosenberg CS, Collins MH, Sette A, Postan M, Tarleton RL 2006 CD8+ T-cell responses to Trypanosoma cruzi are highly focused on strain-variant trans-sialidase epitopes. PLoS Pathog 2:e77. doi:10.1371/journal.ppat.0020077. 16879036 PMC1526708

[47] Amezcua Vesely MC, Schwartz M, Bermejo DA, Montes CL, Cautivo KM, Kalergis AM, Rawlings DJ, Acosta-Rodríguez EV, Gruppi A 2012 FcgammaRIIb and BAFF differentially regulate peritoneal B1 cell survival. J Immunol 188:4792–4800. doi:10.4049/jimmunol.1102070. 22516957 PMC3361506

[48] Quinton LJ, Blahna MT, Jones MR, Allen E, Ferrari JD, Hilliard KL, Zhang X, Sabharwal V, Algul H, Akira S, Schmid RM, Pelton SI, Spira A, Mizgerd JP 2012 Hepatocyte-specific mutation of both NF-kappaB RelA and STAT3 abrogates the acute phase response in mice. J Clin Invest 122:1758–1763. doi:10.1172/JCI59408. 22466650 PMC3336975

[49] Yang XO, Chang SH, Park H, Nurieva R, Shah B, Acero L, Wang YH, Schluns KS, Broaddus RR, Zhu Z, Dong C 2008 Regulation of inflammatory responses by IL-17F. J Exp Med 205:1063–1075. doi:10.1084/jem.20071978. 18411338 PMC2373839

