# Supplementary figures and images for: CD8+ T Cell Immunity Is Compromised by Anti-CD20 Treatment and Rescued by Interleukin-17A
Source: mBio. 2020 May 12;11(3):e00447-20. doi: 10.1128/mBio.00447-20 (PMC7218282; doi:10.1128/mBio.00447-20)

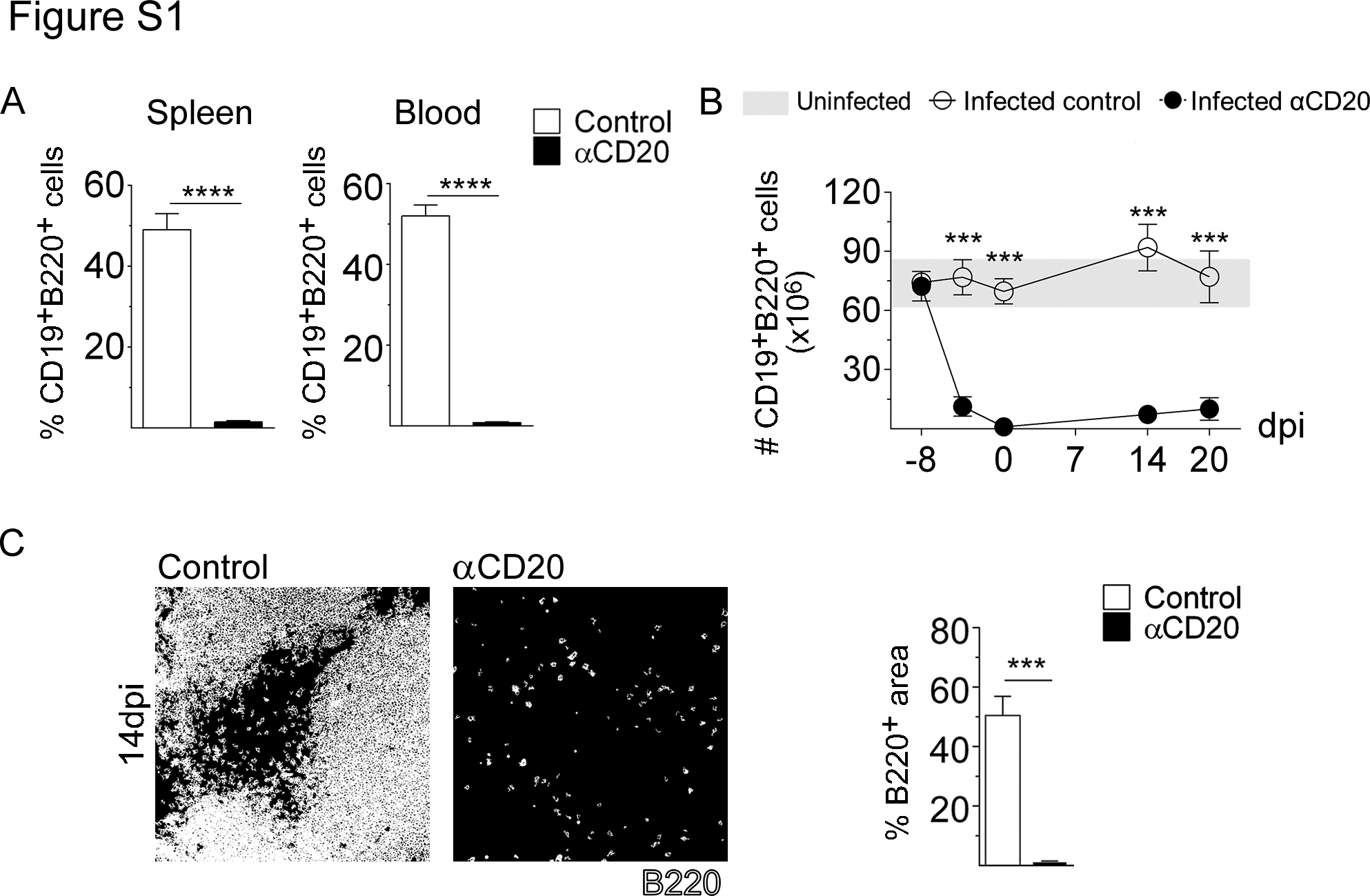

Supplement: FIG S1 [file mBio.00447-20-sf001.tif]

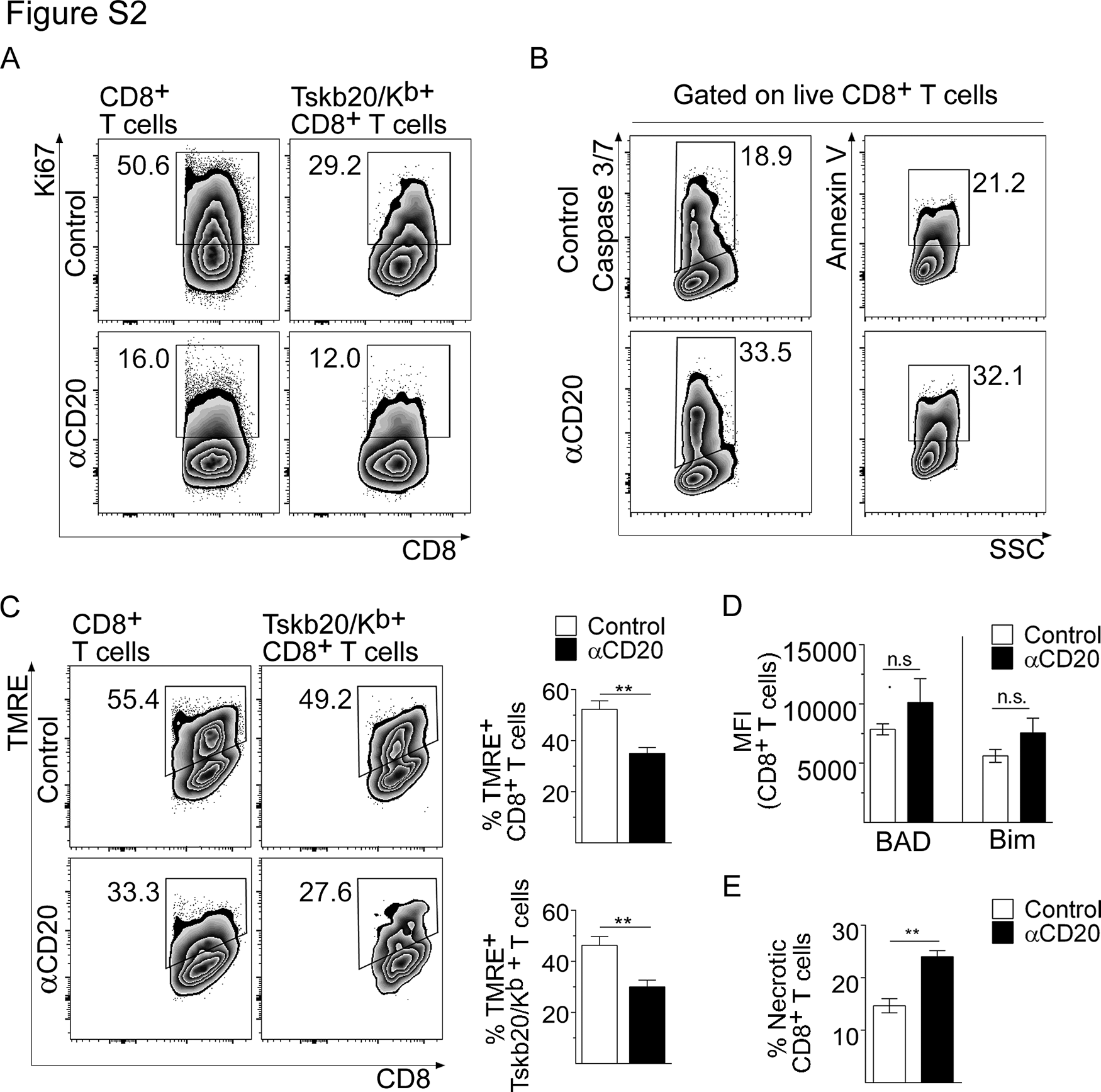

Supplement: FIG S2 [file mBio.00447-20-sf002.tif]

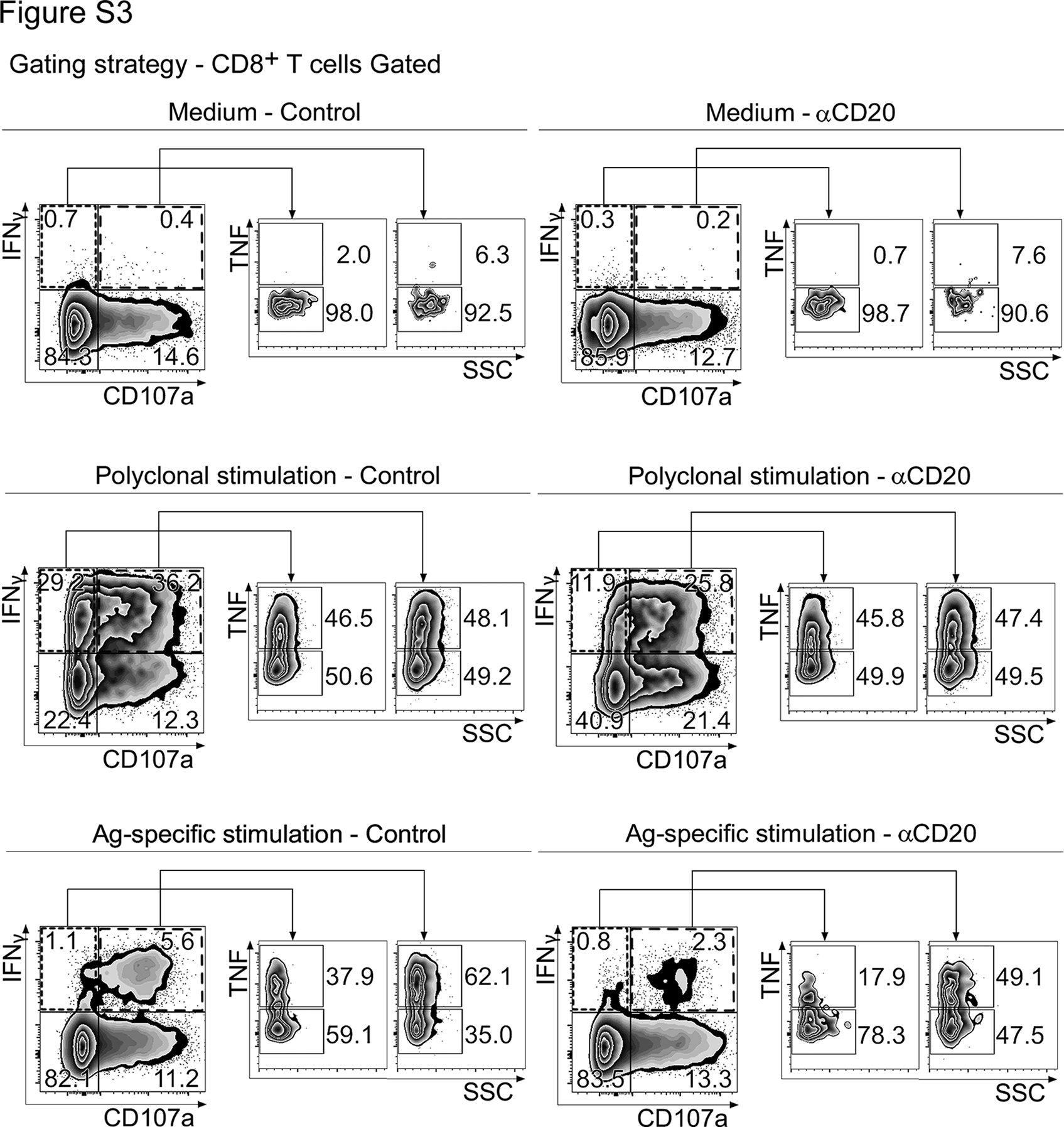

Supplement: FIG S3 [file mBio.00447-20-sf003.tif]

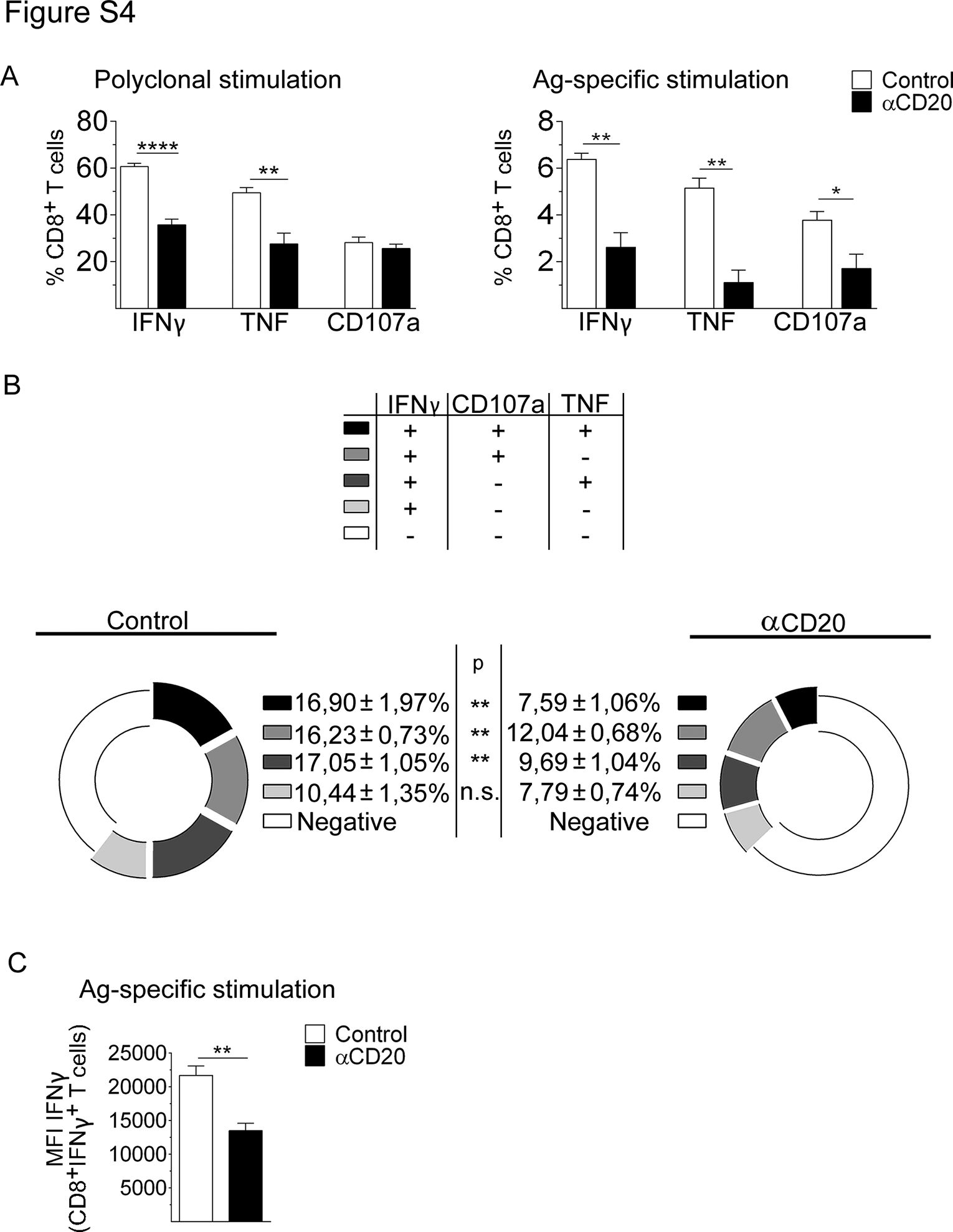

Supplement: FIG S4 [file mBio.00447-20-sf004.tif]

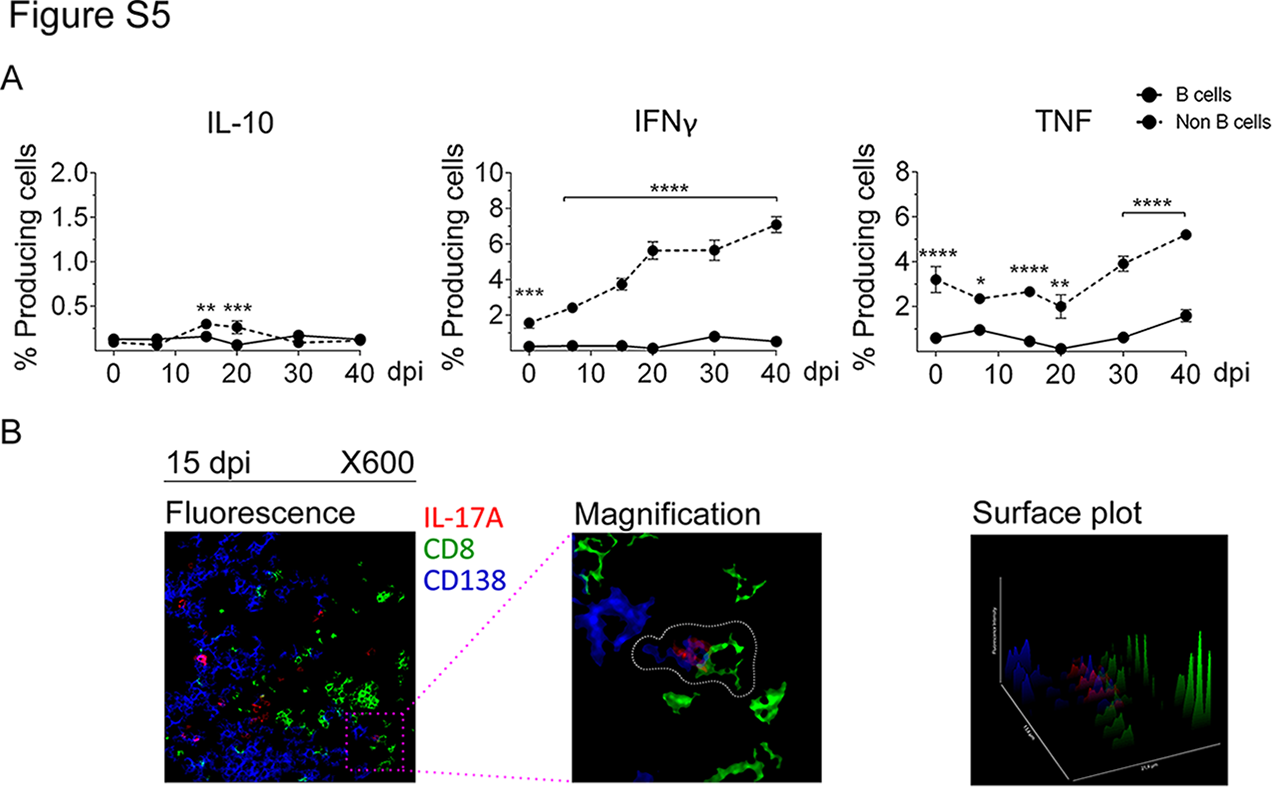

Supplement: FIG S5 [file mBio.00447-20-sf005.tif]

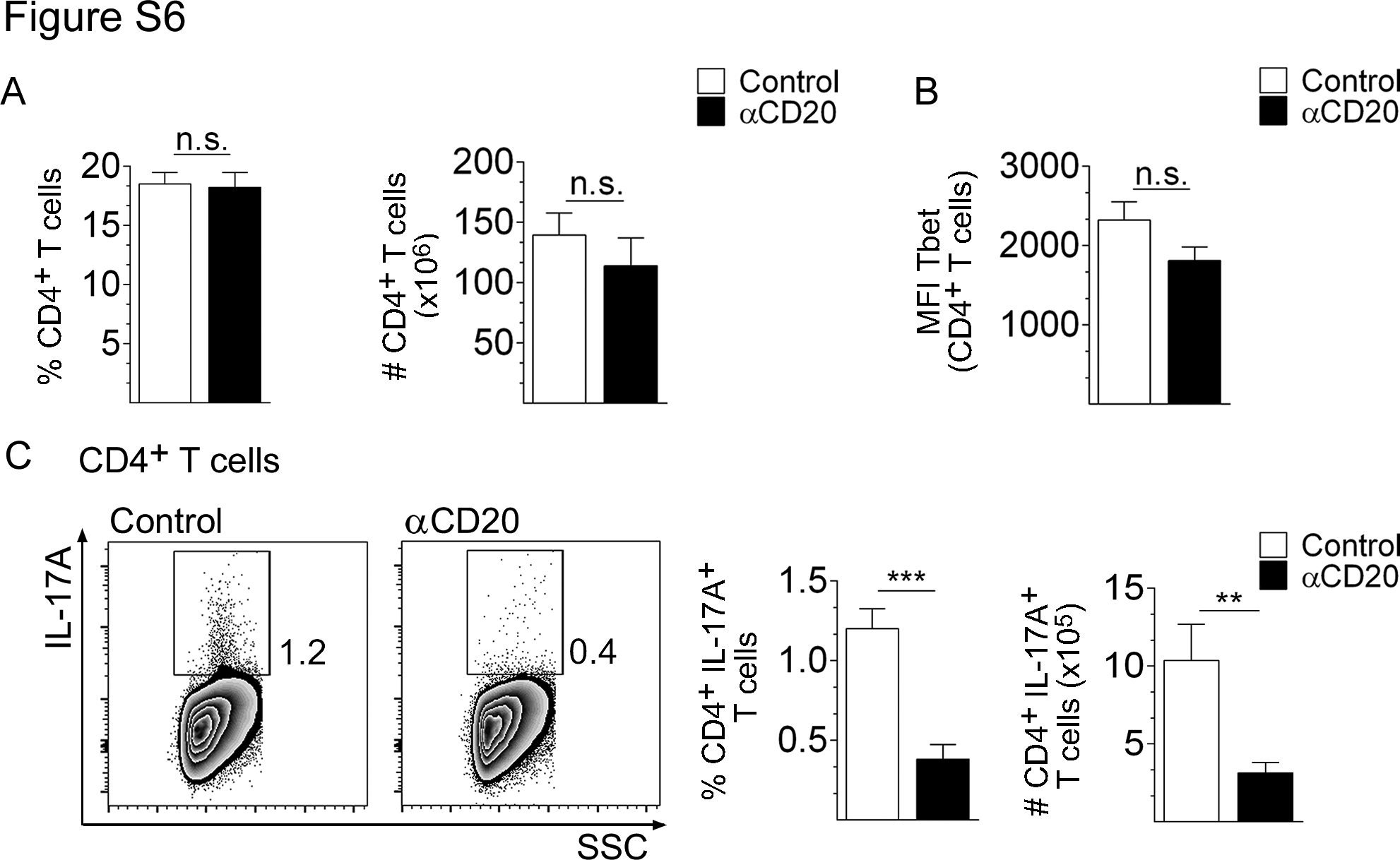

Supplement: FIG S6 [file mBio.00447-20-sf006.tif]
